# Human Platelet‐Derived Extracellular Vesicles Are Internalized by Human Induced Pluripotent Stem Cell‐Derived Neurons Under Control and Hypoxic Conditions

**DOI:** 10.1002/jex2.70168

**Published:** 2026-07-13

**Authors:** Venla Harju, Kai Härkönen, Ulla Impola, Saara Laitinen, Susanna Narkilahti

**Affiliations:** ^1^ NeuroGroup, Faculty of Medicine and Health Technology Tampere University Tampere Finland; ^2^ Blood Service Finnish Red Cross Vantaa Finland

**Keywords:** acute stroke, extracellular vesicles, hypoxia, in vitro, microelectrode array, neuron

## Abstract

Ischaemic brain stroke is among the leading causes of death and disability worldwide. However, the current treatments have a limited time window and regeneration potential. Clinically relevant human platelet‐derived extracellular vesicles (EVs) offer potential neuroprotective treatment for stroke. Here, the neuronal uptake of carboxyfluorescein succinimidyl ester (CFSE)‐labelled EVs was confirmed by confocal imaging and three‐dimensional (3D) image analysis with Imaris software. The results showed that human induced pluripotent stem cell (hiPSC)‐derived neurons can internalize EVs. We also show the colocalization of EVs with cellular organelles: early endosomes and lysosomes. We used an in vitro human model of stroke to study the effects of hypoxia on neurons. After hypoxia, the activity of the neurons, including spiking and bursting, decreased. However, the activity was restored after 72 h of reperfusion. EVs did not affect neuronal activity acutely, but during long‐term follow‐up, neurons showed increased activity. Together, our findings provide insights into the effects of platelet‐derived EVs on neuronal uptake, morphology and functionality and changes after hypoxic insult in an in vitro human model.

## Graphical abstract

Human platelet‐derived extracellular vesicles (EVs) colocalize with cellular organelles, early endosomes and lysosomes, in neurons differentiated from human induced pluripotent stem cells, both in naïve and hypoxic conditions. Furthermore, the functional activity of neurons is changed by hypoxia and by pre‐ and post‐treatment with the EVs in hypoxic conditions.

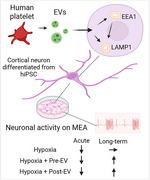

## 1 Introduction

Brain stroke is among the leading causes of death and disability worldwide. Ischaemic brain stroke, the most common type, causes a lack of oxygen and glucose in the stroke core (Mukherjee and Patil 2011). While substantial cell death occurs in the core, the surrounding area, called the penumbra, exhibits cell injury that can potentially be prevented by targeted treatment (Mosconi and Paciaroni 2022). Standard clinical treatment includes the removal of blood clots with thrombolysis and/or thrombectomy, and recovery is supported by rehabilitation in the following months. Many surviving patients are, however, left with permanent morbidities (Virani et al. 2020). Even though a substantial number of clinical trials on neuroprotective treatments have been conducted or are ongoing (Hassan et al. 2024; Paul and Candelario‐Jalil 2021; Pérez‐Mato et al. 2024; Zhao et al. 2024), the translation of these treatments to the clinic has failed. In recent years, extracellular vesicles (EVs) from different cell sources have attracted interest as therapeutic interventions and have been tested in preclinical models of stroke, but large‐scale clinical trials are still needed (Eyileten et al. 2025; Xie et al. 2024; Zhang et al. 2019).

Human cell‐based models are seen as additional tools to animal studies to provide better physiological relevance in evaluating both the safety and efficacy of drugs under development (Sewell et al. 2024). To model stroke in vitro, both oxygen̶‒glucose deprivation (OGD) and hypoxia, with variable parameters ranging from 0.2% to 2% oxygen and from 30 min to 24 h of exposure time, have been used (Cerina et al. 2024; Liu et al. 2020; Otero‐Ortega et al. 2020; Santiago et al. 2023; Voogd et al. 2023). Most human cell‐based in vitro stroke studies thus far have utilized SH‐SY5Y cells from a cancerous neuroblastoma cell line (Voogd et al. 2023) in addition to rodent primary cells and brain slices (Holloway and Gavins 2016). In vitro models of stroke based on human induced pluripotent stem cell (hiPSC)‐derived neurons are still rare but highly promising, as they can potentially offer variable yet defined models with unlimited sources of cells and high human relevance (Nikolakopoulou et al. 2020). Only a few studies have concentrated on the electrophysiological properties of neurons in stroke modelling. Microelectrode arrays (MEAs) provide a means of measuring neuronal network‐level functionality repeatedly and noninvasively (Hyvärinen et al. 2019; Mzezewa et al. 2022). To date, few studies have shown a decrease in neuronal activity in response to hypoxia in hiPSC‐derived neurons; yet, the responses have been reported only in the short term, up to 48 h after hypoxia induction (Cerina et al. 2024; Pires Monteiro et al. 2021).

EVs targeting stroke therapy have been attracted increasing interest in recent years (Hirsch et al. 2023; Jiang et al. 2022; Mirzaahmadi et al. 2025; Thomas et al. 2020; Xie et al. 2024). EVs are heterogeneous vesicles secreted by cells and contain different factors depending on their origin. Additionally, the environment, treatment and activation of EV‐producing cells affect the content of EVs. Importantly, they can cross the blood‐brain barrier (BBB) (Jiang et al. 2022) and penetrate and diffuse into the brain after intranasal administration (Delila et al. 2024). EVs interact either by binding to surface receptors, leading to receptor‐mediated reactions inside the cell, or by internalization followed by cellular uptake of their contents. Uptake occurs by clathrin‐mediated endocytosis (Wang et al. 2025) or by other cell type‐dependent uptake routes (Banizs et al. 2018). Successful internalization of human platelet‐derived EVs by naïve human endothelial cells (Faille et al. 2012), HUVECs (Happonen et al. 2016) and human immune cells (Fendl et al. 2018) has been shown. However, more in vitro internalization studies with different human cell models and three‐dimensional (3D) high‐quality imaging are needed to reveal internalization by particular cell types.

EVs from different sources have been tested as treatments for ischaemia or hypoxia‐reperfusion injury both in vivo and in vitro (Chen and Chopp 2018; Leiter and Walker 2019; Zhang et al. 2019). For example, as a neuroprotective treatment for ischaemia in vitro, EVs derived from human embryonic stem cell (hESC)‐derived neural progenitor cells (NPCs), astrocytes and neurons have been administered to hESC‐derived neurons (Deng et al. 2018), and human neural stem cell (hNSC)‐EVs have been administered to hNSCs (Liu et al. 2020). Human blood is an interesting source of EVs extracted from platelets, and blood‐derived samples have already been validated for clinical use. Thus, blood‐derived EVs would not require any additional regulatory permissions or operations for collection for therapeutic purposes. Different methods to generate human platelet lysates (HPLs) or to isolate EVs from platelet concentrates (PCs) pooled from otherwise discarded blood donations have been described in recent work (Yeh et al. 2024). Preclinical research has already shown the therapeutic potential of HPLs for treating various central nervous system diseases (Nebie et al. 2022), including stroke (Hayon et al. 2013). Platelet EVs are important for this process (Nyam‐Erdene et al. 2021), as they contain many neurotrophic factors, anti‐inflammatory proteins and antioxidants (Delila et al. 2024). A review of clinical trials of EVs revealed that EV therapy is safe, but its efficacy needs to be studied further, and standardization of the reporting EV isolation and characterization is needed (Van Delen et al. 2024). Despite these promising results, more studies with human‐relevant models, such as hiPSC‐based neuronal stroke models, are needed to elucidate the usability of EV therapy for treating stroke.

Here, the internalization of platelet‐derived EVs into hiPSC‐derived neurons was evaluated in detail and quantified with image analysis tools. The possible cytotoxicity to neurons and effects of EVs on the morphology and functionality of neurons were studied. Furthermore, a hypoxic insult was introduced to the neuronal networks, and its effect on EV internalization was studied. Resulting hypoxic injury to neuronal networks was evaluated at the morphological and functional levels during the acute and recovery periods up to 2 weeks. Finally, potential treatment involving EVs and its effect on the response of neuronal networks to hypoxia was studied. Here, we highlight the need for longer follow‐up and functional testing for in vitro models of human stroke.

## 2 Materials and Methods

### 2.1 HiPSCs and Neuronal Differentiation

UTA.04511.Wts hiPSCs (Ojala et al. 2016, registered to https://hpscreg.eu/), obtained from the Faculty of Medicine and Health Technology (MET), iPS Cells Facility, Tampere University, Finland, was used for neuronal differentiation. Supportive statement from the Ethics Committee of the Expert Responsibility area of Tampere University Hospital to be used in neuronal research (R20159) exists. The cell line is under quality control with frequent gene and protein expression analyses for pluripotency, karyotype normality, and mycoplasma.

The hiPSCs were transferred to feeder‐free culture on the wells coated with 15 µg/mL human recombinant laminin‐521 (LN521, BioLamina, Sundbyberg, Sweden) in E8 medium (Thermo Fisher Scientific, Waltham, MA, USA), as previously described (Hongisto et al. 2017). Human iPSCs were differentiated into cortical neurons according to a previously published protocol (Hyvärinen et al. 2019). The differentiation protocol included neuronal induction, precursor expansion, and maturation phases, which lasted 32 days in total.

After the differentiation, the cells were plated for cell experiments; this day is referred to as days in vitro (DIV) 0. Cells were plated on either 48‐well glass bottom plates (MatTek Life Sciences, Ashland, MA, USA) for confocal imaging, 48‐well plastic bottom plates (Thermo Fisher Scientific) for cell viability assays or 48‐well MEA plates (Axion CytoView MEA 48, Axion BioSystems, Atlanta, GA, USA) for functional studies. For glass and plastic wells, cells were plated at a density of 50 000 cells/cm^2^ to wells coated with 100 µg/ml poly‐L‐ornithine (PLO, Sigma‐Aldrich, Saint Louis, MO, USA) in 0.1 M borate buffer and 15 µg/ml LN521. For the MEA plates, 80 000 cells were plated in a 10‐µl droplet (635 000 cells/cm^2^). MEA plates were coated with a 10‐µl droplet of 0.1% polyethylenimine (PEI, Sigma‐Aldrich) in 0.1 M borate buffer followed by a 10‐µl droplet of 50 mg/ml LN521.

From DIV 0 onwards, neural maturation medium (NMM), consisting of a 1:1 mixture of D‐MEM/F12 (with GlutaMAX) and neurobasal medium supplemented with 0.5% N2, 1% B27 with retinoic acid, 0.5 mM GlutaMAX, 0.5% nonessential amino acid solution, 50 µM 2‐mercaptoethanol, 0.1% penicillin/streptomycin (all from Thermo Fisher Scientific), 2.5 µg/mL insulin (Sigma‐Aldrich), 20 ng/mL brain‐derived neurotrophic factor (BDNF, R&D Systems, Minneapolis, MN, USA), 10 ng/mL glial‐derived neurotrophic factor (GDNF, R&D Systems), 500 µM dibutyryl‐cyclic AMP (db‐cAMP, Sigma‐Aldrich), and 200 µM ascorbic acid (AA, Sigma‐Aldrich), was used. For the neurons cultured on MEA plates, BrainPhys maturation media, consisting of BrainPhys neuronal medium (Stemcell Technologies, Vancouver, Canada) supplemented with 0.5% N‐2, 1% B‐27 with retinoic acid, 0.1% penicillin/streptomycin, 20 ng/mL BDNF, 10 ng/mL GDNF, 500 µM db‐cAMP and 200 µM AA, was used. At DIV 0 during plating, 10 µM ROCK inhibitor (Sigma‐Aldrich) was added to the medium to support cell survival. The cultures were maintained at +37°C in a 5% CO_2_ atmosphere and 95% humidity, and the media was changed three times a week.

### 2.2 Platelet Concentrate Preprocessing

PCs not needed for clinical use were obtained from clinically relevant human blood samples by the Finnish Red Cross Blood Service (Vantaa, Finland). PCs were prepared by combining four buffy coats. PCs were leuco‐reduced, not irradiated and they were stored at room temperature (RT) under constant agitation. All donated blood products used for research were from healthy donors who had provided informed consent. All blood products were treated anonymously. The research was in accordance with the rules of the Finnish Supervisory Authority for Welfare and Health (Valvira, Helsinki, Finland). Research permission was also obtained from the local Blood Service Board (Finnish Red Cross Blood Service, Finland).

### 2.3 Isolation of EVs

PC was diluted 1:2 with Dulbecco's phosphate‐buffered saline (DPBS) supplemented with 111:1000 of acid citrate dextrose (Terumo BCT, Lakewood, CO, USA) and centrifuged for 7 minutes (min) at 650 × g at RT without braking and again at 1560 × g for 20 min at RT with an Eppendorf 5810R (Eppendorf, Hamburg, Germany). The supernatants were then filtered using SteriCup Filter Units with a 0.22‐µm pore size (Merck KGaA, Darmstadt, Germany). The resulting cell and debris‐free supernatants were further centrifuged first at 100 000 × g for 1.5 hours (h) at +4°C with Optima MAX‐XP Ultracentrifuge with a MLA‐50 rotor (k‐factor 92, Beckman Coulter, Brea, CA, USA), washed with DPBS, and centrifuged again at 100 000 × g for 1.5 h at +4°C (in an MLS‐50 rotor, k‐factor 71). The resulting platelet EV pellets were carefully suspended in 200 µL of DPBS and centrifuged again at 3000 × g for 10 min to remove any larger aggregates. The supernatant was collected, aliquoted and stored at +4°C until use.

### 2.4 Characterization of EVs

Characterization of similar EVs has been performed previously (Ilvonen et al. 2024; Palviainen et al. 2024). Particle concentration and size distribution of platelet EV samples were analyzed with the nanoparticle tracking analysis (NTA) instrument ZetaView PMX‐120 (ParticleMetrix). Particle size distribution is shown in Figure S1.

EV samples containing 5 µg of protein (analyses with NanoDrop Spectrophotometer) were diluted in Laemmli buffer (Bio‐Rad) and loaded onto Mini‐Protean TGX 4%–20% gels (Bio‐Rad) and run at 40 V for 10 min and 200 V for 35 min in 1 × Tris/Glycine/SDS Buffer (Bio‐Rad). Trans‐Blot Turbo 1×TransBlot Transfer Buffer with 20% ethanol and TransBlot Turbo Mini‐size PVDF Membrane (all from Bio‐Rad) were used for semi‐wet transfer. The membrane was blocked in 3% BSA (Biowest). Primary antibodies against CD9, CD63, ApoA1 and ApoB (Medix Biochemica) were prepared at a 1:1000 dilution in TBS T containing 3% BSA (TBS with 0.1% Tween 20). The membranes were incubated with these antibodies overnight at +4°C, followed by three 10‐min washes in TBS T.

For detection, the secondary antibodies Goat Anti‐Mouse IgG and Goat Anti‐Rabbit IgG (Bio Rad) were diluted 1:500, while the Streptactin HRP Conjugate (Bio Rad) was diluted 1:5000, all in TBS T. Membranes were exposed to the secondary reagents for 1 h at RT and then washed again three times for 10 min in TBS T, with an additional final 5 mi rinse in PBS. After washing, Clarity Western ECL Substrate (Bio Rad) was applied to the membranes, and the resulting chemiluminescent signal was captured using the ChemiDoc Touch Imaging System (Bio Rad).

The origin and purity of platelet EVs was verified with Amnis ImageStream X Mark II analysis, as EVs were labelled for 60 min at RT in the dark using antibodies against erythrocyte Glycophorin A (CD235a, clone: HIR2, BD Pharmingen), CD9 (clone: MEM‐61, Exbio) and platelet specific marker CD41 (clone: HIP8, BioLegend). Samples were negative for CD235 and 98% positive for CD41. Possible CD9 antibody aggregates were eliminated by diluting the antibodies first 1:15 in DPBS and centrifuging them at 16000 × g for 10 min after which the supernatants were used for labelling. Sample buffer and antibody‐only samples were used for background labelling. Labelled EV samples were analysed using an Amnis ImageStreamX Mk II imaging flow cytometer (Cytek Biosciences). Detection was performed with 488 nm, 642 nm, and 785 nm excitation lasers, using the Amnis High Gain setting and a 60 × objective. The collected data were processed and evaluated using IDEAS Analysis Software version 6.4.

### 2.5 EV Labelling With Carboxyfluorescein Succinimidyl Ester

Carboxyfluorescein succinimidyl ester (CFSE, Merck) was used to label the EVs and estimate their total number at a wavelength of 488 nm. Labelling was performed as previously described (Ilvonen et al. 2024). Briefly, 10E+10 EVs/mL were incubated with 20 µM CFSE in a total volume of 100 µL in the dark at 37°C for 15 min. The excess CFSE was removed using resin columns according to the manufacturer's instructions (Thermo Fisher Scientific). Samples with CFSE without EVs (mock controls) were performed in parallel fashion to confirm the free dye retention by columns. Labelled EVs were analysed with an Amnis ImageStreamX Mk II flow cytometer (Luminex/Cytek Biosciences, Fremont, CA, USA). CFSE‐labelled EVs were used for compensation, and unlabelled EVs and mock controls were used to determine the autofluorescence and background noise.

### 2.6 Addition of EVs to Neuronal Cultures

EVs were vortexed for 30 seconds (sec) before they were diluted in cell media. Next, 10E+10 EV particles diluted in 10 µL of medium were added to every well in a 48‐well plate containing 500 µL of media in total. Amount of used particles was selected based on previous works (Auber and Svenningsen 2022; Nieuwland and Siljander 2024). Each dilution was triturated before it was added to the wells and then mixed with the cell media after it was added to the wells. The EV/cell ratio was 125 000/cell on MEA plates and 700 000/cell on other plates according to calculations based on the numbers of plated cells. Additionally, amounts of 10E+9 and 10E+11 EVs/well were tested (Figure S1) before the EV uptake experiments were performed to confirm that a reasonable number of EVs reached the cells. CFSE mock controls without EVs were used without dilution. The incubation times tested were 1and 8 h for the uptake experiments and 24 h for the functional studies. Uptake experiments were performed at DIV 14, and functional studies were performed at DIV 32.

### 2.7 Immunocytochemical Staining

Immunocytochemical (ICC) staining was performed as previously described (Lappalainen et al. 2010; Mäkinen et al. 2013). Briefly, cells were fixed with 4% paraformaldehyde (PFA) for 15 min, washed twice with PBS and blocked with 10% normal donkey serum (NDS, Merck), 0.1% saponin (S7900, Sigma‐Aldrich) and 1% bovine serum albumin (BSA, Sigma‐Aldrich) in PBS at RT for 45 min. After blocking, the samples were washed with primary washing solution containing 1% NDS, 0.1% saponin and 1% BSA in PBS. The samples were then incubated overnight at +4°C with primary antibodies in washing solution. The primary antibodies used were anti‐βtub_III_ (ab41489 chicken (chk), 1:200, Cell Signalling Technology, Danvers, MA, USA), anti‐MAP2 (NB300‐213 chk, 1:400, Novus Biologicals, Minneapolis, MN, USA), anti‐EEA1 (ab70521 mouse (ms), 1:1000, Abcam, Cambridge, UK), anti‐LAMP1 (H5G11 SC‐18821 ms, 1:100, Santa Cruz Biotechnology, Dallas, TX, USA), anti‐LAMP1 (ab25630 ms, 1:500, Abcam), and anti‐Cleaved Caspase‐3 (Cl‐Casp3, 9664L rabbit, 1:400, Cell Signalling Technology). Then, the samples were washed twice with secondary washing solution containing 1% BSA and 0.1% saponin in PBS, after which they were incubated at RT for 1 h with secondary antibodies in the same solution. The Alexa Fluor‐labelled secondary antibodies used were goat anti‐mouse 568 (A21043, 1:400), goat anti‐chicken 568 (A11041, 1:400), donkey anti‐rabbit 568 (A10042, 1:400), and goat anti‐chicken 647 (A21449, 1:200) (all from Thermo Fisher Scientific). Finally, the samples were washed with PBS, incubated for 30 min with DAPI (D9542, 1:4000, Sigma‐Aldrich) in PBS and washed with PB before they were mounted with VECTASHIELD Antifade Mounting Medium either with or without DAPI (Vector Laboratories Inc., Newark, CA, USA). Samples were fixed at 1 or 8 h after treatment.

### 2.8 Image Analysis

Images were captured with a Zeiss Axio Observer and a Z1 inverted LSM 800 laser scanning confocal microscope (Carl Zeiss AG, Oberkochen, Germany). For neuronal morphology images, a Zeiss LD LCI Plan‐Apochromat 25× multi‐immersion objective (numerical aperture (NA) 0.8) with glycerol was used. For images of the cellular internalization of EVs, a Zeiss Plan‐Apochromat 63× oil immersion objective (NA 1.40) was used. After imaging, 3D image stacks were deconvolved with Huygens Essential software (Scientific Volume Imaging, Hilversum, Netherlands). Analysis of EV localization was performed with Imaris Single Full Image Analysis Software versions 10.1.0 and 10.2.0 (Oxford Instruments plc, Abingdon, UK).

Analysis of neuronal networks was performed using Imaris with the “Surface” function, as the “Filament” function was not optimal for detecting neurites here. The “Filament” function attempts to connect every neurite to its own neuron, while here, some neuronal structures were connected to neurons with somas and nuclei outside the image. Thus, the “Surface” function worked better with our samples.

For EV internalization analysis, EVs were detected with both the “Spots” and “Surface” functions, whereas cells and organelles were detected with the “Surface” function. The “Surface” function builds a surface object around the structures of interest in a particular channel based on the channel's intensities that correspond to those structures. The segmentation performed by Imaris includes more parameters than absolute intensity. The background subtraction method was used for thresholding, including calculations for local intensity landscapes. Segmentation was performed with different thresholds to different channels and tuned with the help of control images so that the autofluorescence of the structures was not detected as a signal (autofluorescence shown in Figure S1). Both EV and cellular organelle surface channels were then filtered such that the “shortest distance to the neuronal channel < 0”, resulting in populations inside and outside the neuronal surface. Given that the MAP‐2 + βtub_III_ channel represents filaments inside the cell membrane of the neurons, the EVs touching the neuronal channel were considered to be inside the neuron. Similar filtering was then performed for the EVs with “shortest distance to the EEA1/LAMP1 channel < 0” to obtain the populations that were either touching/colocalizing with the cellular organelle surfaces or not. Examples of image analysis steps are shown in Figure S2.

The quantified parameters used in this study are as follows:

EV uptake = number of EVs inside the neuronal surface reconstruction/total number of EVs in the image (percentage of spots)
EV volume fraction = total volume of EV surface reconstruction inside the neuronal surface/total volume of the neuronal surface reconstruction in the image
EV coloc EEA1 or LAMP1 = number of EV surfaces within distance < 0 from the EEA1 or LAMP1 surface/total number of EV surfaces inside the neuron (percentage of surfaces)
Sphericity = the ratio of the surface area of a sphere (with the same volume as the given particle) to the surface area of the particle.

### 2.9 Cytotoxicity Assays

The Colorimetric Lactate Dehydrogenase (LDH)‐Cytotoxicity Assay Kit (ab65393, Abcam) was used according to the manufacturer's instructions to assess the potential cytotoxicity of the EVs under naïve or hypoxic conditions. Briefly, the conditioned media from the cell samples was collected, centrifuged and stored at –80°C before the assay was conducted. The fluorescence intensity was immediately measured with a Viktor Nivo microplate reader (PerkinElmer, Waltham, MA, USA) at an absorbance of 450 nm and a controlled temperature of +37°C in kinetic mode every 3 min for 60 min. Each sample was analysed in technical duplicate. Samples were collected 8, 24 and 72 h after the addition of EVs, after 8 and 24 h of hypoxia, and after 48 h of reperfusion, and parallel timepoints for control conditions were used. The results were normalized to those of baseline samples collected 24 h before the treatments.

To study cell viability, LIVE/DEAD assay was performed according to the manufacturer's instructions. Neurons incubated with EVs for 24 h were stained with 0.1 µM Calcein‐AM (Invitrogen, C3099) and 0.5 µM EtHD‐1 (Invitrogen, E1169) for 30 min. Cells were imaged with Olympus IX51 microscope (Olympus Corporation, Japan) and number of live and dead cells were analyzed with Imaris software. Number of apoptotic cells positive for Cl‐Casp3 were analyzed using colocalization of reconstructed surfaces of DAPI and Cl‐Casp3.

### 2.10 MEA Measurements

Extracellular neuronal network activity was recorded with an Axion Maestro system controlled by AxIS Software (Axion Biosystems) with a sampling rate of 12.5 kHz and under a controlled temperature of 37°C as previously described (Hyvärinen et al. 2019). Prior to all the recordings, the MEA plates were allowed to equilibrate for 5 min. The development of spontaneous activity was recorded twice a week for 10 min until treatment with EVs or hypoxia.

For hypoxia measurements, the media was changed one day prior to the baseline recording for both hypoxia treated and control wells. The activity was recorded before the 24‐h hypoxia period and immediately after hypoxia. The media was subsequently changed to allow a 24‐h reperfusion period before the first reperfusion recording. The second reperfusion recording was collected after 72 h of reperfusion. Then, the activity of the cultures was recorded twice a week for two weeks.

### 2.11 MEA Data Analysis

Spike detection was performed using a previously described method (Lieb et al. 2017; Mayer et al. 2018) embedded in an in‐house‐made MATLAB (MathWorks, Natick, MA, USA) script (Hyvärinen et al. 2019). A threshold value of 4.5 × the estimate of the noise standard deviation was used for amplitude thresholding (Quiroga et al. 2004). Only the spikes detected by both algorithms were considered true positive, and only the electrodes detecting >10 spikes per min were considered active. Bursts were identified at the single electrode level from the spike timestamps with the logISI algorithm (Pasquale et al. 2010), which revealed local population activity. The minimum number of spikes per burst was set to five, and the cut‐off for the interspike interval (ISI) and the default maximum ISI (maxISI) were set to 100 ms. Spike and burst features as well as other MEA parameters were computed with meaRtools and averaged per well (Gelfman et al. 2018). Burst features were presented only for electrodes detecting bursts at the baseline timepoint. If no bursts were detected after the baseline, the electrode was excluded, but if the bursting activity was recovered at the later follow‐up time points, the number of bursts was set at 0 for the timepoints when it was absent.

### 2.12 Hypoxia and Reperfusion

For functional and EV uptake experiments, hypoxic conditions were used. The cells were incubated at 37°C in a 1% O_2_, 5% CO_2_ atmosphere for 24 h, as previously reported (Juntunen et al. 2020) in BrainPhys maturation media. The media was changed 24 h prior to the cells being subjected to hypoxic conditions. After hypoxia, samples were collected, and recordings were performed under normal atmospheric conditions, after which the media was changed, and the reperfusion period commenced for the functional experiments. The media was changed to control conditions at the same time points as those used for the hypoxia samples.

### 2.13 Statistical Analysis

Statistical analyses were performed using GraphPad Prism software (v 10.3.1 GraphPad, Boston, MA, USA). All the data did not follow a normal distribution; thus, nonparametric tests were used. The sample sizes of the different experiments are listed in Table S2 and in the respective figure legends. EV uptake and neuronal morphology quantification data are presented as truncated violin plots, extending from minimum to maximum values, with dashed lines showing each quartile and dots showing individual data points. LDH data were normalized to baseline measurements and are shown as the median ± 95% confidence interval (CI). MEA data are presented as the mean ± 95% CI or as box plots with box borders for the 25th and 75th percentiles, whiskers for the minimum to maximum values, the middle line for the median and dots for the individual data points. If the MEA data were normalized, normalization was performed to the baseline measurement (100%). All comparisons between two groups were performed using the nonparametric Mann‒Whitney U test. Comparisons among three groups were conducted using the Kruskal‒Wallis test with Dunn's correction for multiple comparisons. Normalized percentile values compared to baseline values were analysed using the Wilcoxon signed‐rank test.

Statistical significance is denoted in the figures as *p < 0.05, **p < 0.01, ***p < 0.001, and ****p < 0.0001, and the exact *p* values are listed in Table S1.

## 3 Results

### 3.1 Human iPSC‐Derived Neurons Internalize EVs

**FIGURE 1:**
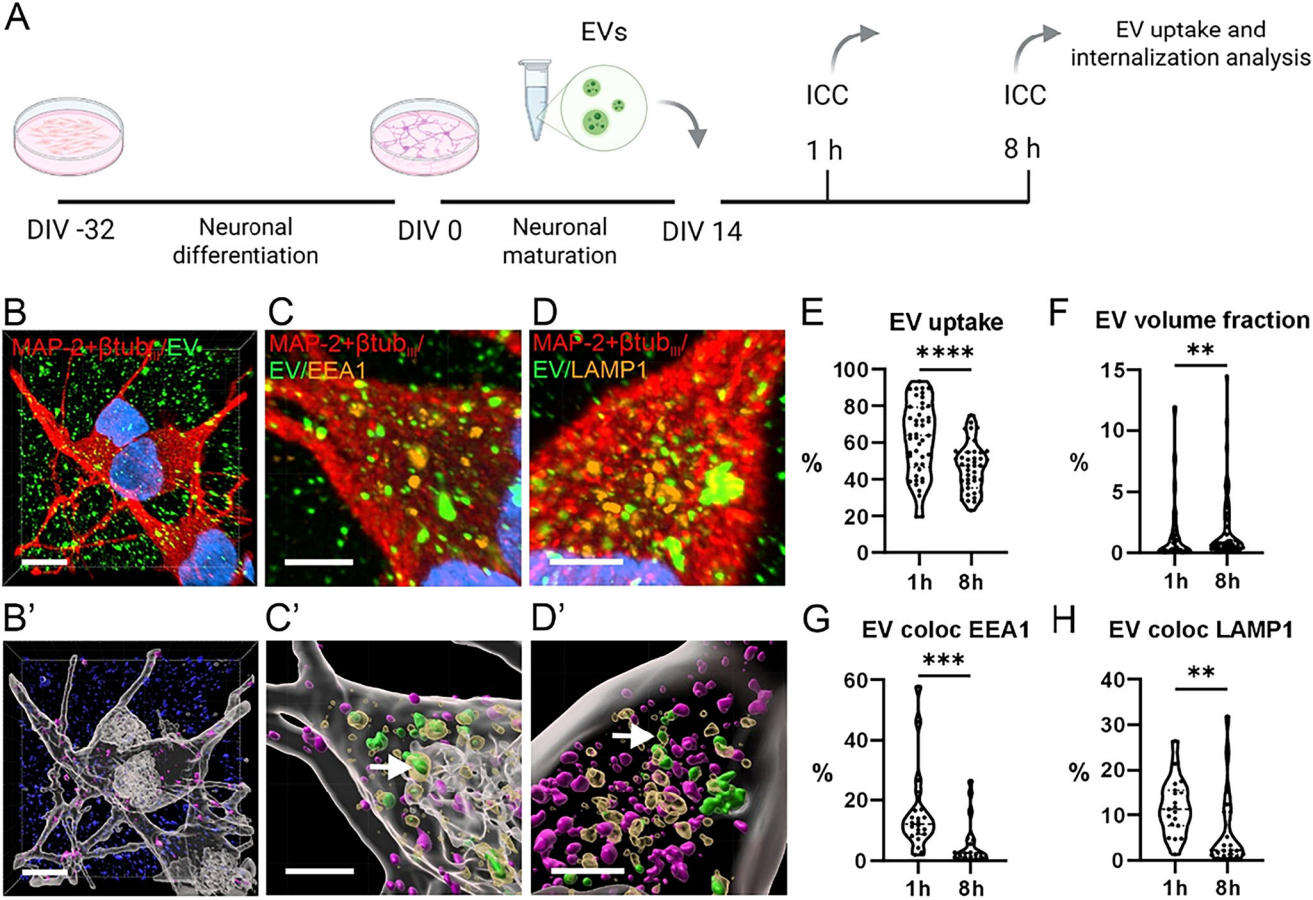
Internalization of EVs into neurons. A) Timeline and graphical representation of the experiment. Created in BioRender. Harju, V. (2025) https://BioRender.com/zdlgy88. B) Immunostained and B’) reconstructed images of EVs and neurons. The scale bar is 10 µm. DAPI was used to stain the nuclei of the cells (blue), while neurons were co‐labelled with MAP‐2 + βtub_III_ (red) and EVs with green fluorescent protein (CFSE) in B, C and D. In the reconstructed image B’, the EVs are blue when outside neurons (shown as a white surface) and violet when inside neurons. C) Immunostained and C’) reconstructed images of EVs and their colocalization with EEA1 inside neurons. The scale bar is 3 µm. In C, EEA1 staining is shown in orange, and in C’, EEA1 is shown in yellow; EVs inside neurons not colocalized with EEA1 are shown in violet, and EVs colocalized with EEA1 are shown in green, as indicated by the arrow. D) Immunostained and D’) reconstructed images of EVs and their colocalization with LAMP1 inside neurons. The scale bar is 3 µm. In D, LAMP1 staining is shown in orange, and in D’, LAMP1 is shown in yellow; EVs inside neurons not colocalized with LAMP1 are shown in violet, and EVs colocalized with LAMP1 are shown in green, as indicated by the arrow. E) Quantification of EV uptake at 1 and 8 h. F) Quantification of the total volume of EV surfaces inside neurons divided by the neuronal volume. Uptake results (E&F) are from two experiments, and each point represents one image (*n* = 43–47) taken from thirteen to fourteen individual cell cultures per time point. The statistical significances were calculated with the Mann‒Whitney U test. **p < 0.005, ****p < 0.0001. G) Quantification of the colocalization of EVs with EEA1 at 1 and 8 h. H) Quantification of the colocalization of EVs with LAMP1 at 1 and 8 h. The cellular organelle results (G&H) are from two experiments, and each point represents one image (*n* = 18–22) taken from eight individual cell cultures. The number of samples and EV surfaces included in the colocalization results are shown in Table S2. The statistical significances were calculated with the Mann‒Whitney U test, **p < 0.005, ***p < 0.0005. The results in E‐H are presented as truncated violin plots, extending from minimum to maximum values, with dashed lines showing each quartile and dots showing individual data points. EV = extracellular vesicle, ICC = immunocytochemical staining, DIV = days in vitro, EEA1 = early endosome antigen 1, LAMP1 = lysosomal‐associated membrane protein 1.

The internalization of EVs by human neurons under control conditions was first evaluated. CFSE‐labelled EVs were added at a concentration of 2*10E+10 vesicles/ml to neurons and their internalization was followed for 1 and 8 h (Figure 1A). Using an optimized ICC staining protocol with saponin as a cell‐permeability inducer, followed by confocal imaging, and analysis with Imaris software, the number and volume of CFSE‐stained EVs localized within neuronal surfaces and colocalized with cellular organelles, namely, early endosomes and lysosomes, were visualized and quantified. First, the internalization of EVs into neurons was successful (Figure 1B,B’). The quantification of EV number from the reconstructed images revealed a decrease of internalization ratio from 64% to 47% from 1 to 8 h (Figure 1E). The EV internalization was further studied by calculating portion of the EV volume relative to the total neuronal volume, which increased from 0.3% to 0.8% during the 8‐h follow‐up (Figure 1F). Of those internalized EVs, 12% colocalized with an early endosome marker (EEA1) at 1 h time point decreasing thereafter to 3% at 8 h (Figure 1C,C’,G). Similarly, EVs showed decreasing colocalization with a lysosome marker (LAMP1) from 1 to 8 h (11% to 2%, respectively, Figure 1D,D’,H). Thus, CFSE‐labelled EVs can be internalized by human neurons under control conditions and colocalize with expected cellular organelles with higher rate of colocalization in earlier time point.

### 3.2 EVs Have a Minor Influence on Cytotoxicity, Neuronal Morphology and Functionality Under Control Conditions

**FIGURE 2:**
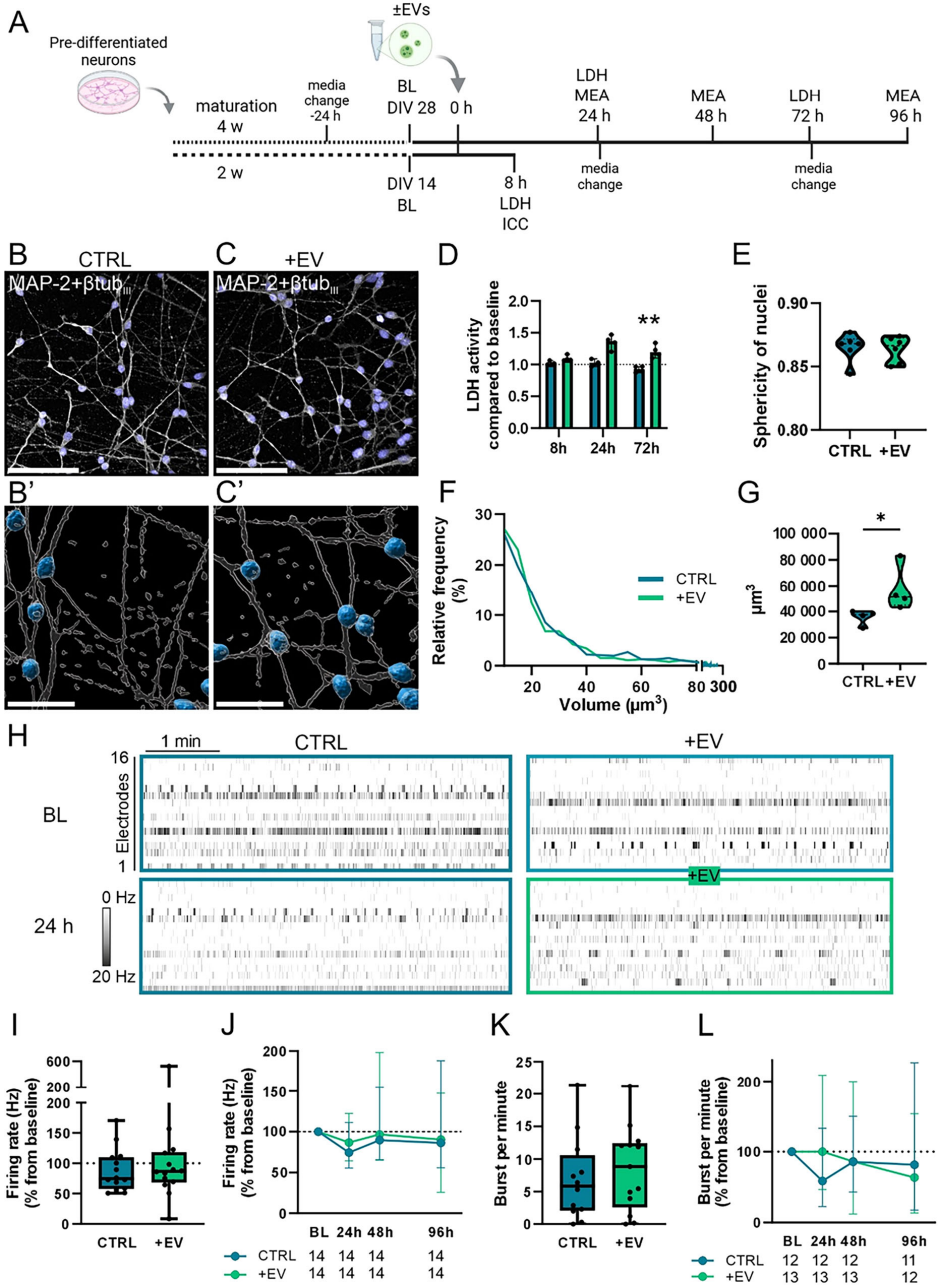
Effects of platelet‐derived EVs on neurons under control conditions. A) Timeline and graphical representation of the experiments. Created in BioRender. Harju, V. (2025) https://BioRender.com/vmi7809. B) Staining and B’) reconstruction of neuronal networks without EVs. C) Staining and C’) reconstruction of the neuronal networks cultured 8 h with EVs. In B, B’, C and C’, nuclei were stained with DAPI (blue), and neurons were co‐labelled with MAP‐2 and βtub_III_ (white). The scale bar in B&C is 100 µm and in B’&C’ 30 µm. D) LDH activity compared to baseline with or without EV treatment at 8, 24 or 72 h. The results are from one experiment, each sample (*n* = 4 for every group) pooled from two wells, **p < 0.005. Statistical significances were calculated with the Kruskal‒Wallis test with Dunn's multiple comparisons. Results are shown as the median ± 95% CI. E) The sphericity of neuronal nuclei with or without EV treatment. F) The volumes of neuronal surfaces with or without EV treatment. The volumes and sphericity results are from one experiment, and each point represents one image (*n* = 4) taken from two to four individual cell cultures, *p < 0.05. Statistical significances were calculated with the Mann‒Whitney U test. The results in E and F are presented as truncated violin plots, extending from minimum to maximum values, with dashed lines showing each quartile and dots showing individual data points. G) The frequency distribution of neuronal volumes under control conditions and after 8 h treatment with EVs. H) Representative raster plots of neuronal activity measured with MEA during baseline (prior EV add) and after 24 h in control and EV‐treated conditions. I) Mean firing rate of neurons in control conditions and after 24 h treatment with EVs. J) Mean firing rate of neurons following the treatment for four days with media changes in between. K) Bursts per minute in control conditions and after 24 h treatment with EVs. L) Comparison of the burst rate to baseline (100%) four days following the EV treatment and in control conditions. The MEA results are from one experiment, *n* = 14 wells for both control and EV treatment. Data in I and K are presented as box plots with box borders for the 25th and 75th percentiles, whiskers for the minimum and maximum values, the middle line for the median and dots for the individual values. Data in J and L are presented as the mean ± 95% CI. Wilcoxon signed‐rank test revealed no significant differences between baseline (100%) and conditions in different timepoints. MEAs presenting bursting activity at baseline were included to analysis. If there were no bursts detected after the baseline measurement, the electrode was excluded, but if the bursting activity was recovered during the follow‐up period, the number of bursts was set at 0 for the timepoints when absent. The numbers of samples for each comparison and timepoint are shown in Table S2. EV = extracellular vesicle, BL = baseline, DIV = days in vitro, LDH = lactate dehydrogenase, MEA = microelectrode array, ICC = immunocytochemical staining.

As EVs from different sources have shown either neurotoxicity or neuroprotection (Oyarce et al. 2022), cytotoxicity and morphological analysis were performed in the neuronal cultures at DIV14 (Figure 2A). After 24 h treatment with platelet‐derived EVs, no differences were found in percentage of apoptotic cells or number of live or dead cells (Figure S3). Also, no metabolic effect of EVs was observed after 8 h treatment when compared with controls when measured with LDH assay (Figure 2D, *p* > 0.05). Analysis of nuclei sphericity revealed no differences, either (Figure 2E, *p* > 0.9999). Furthermore, compared with that of the controls, the morphology of the ICC‐stained and reconstructed neuronal networks appeared similar (Figure 2B,C,B’,C’, Figure S3), as did the frequency distribution of the neuronal surface volume (Figure 2F). More detailed morphological analysis revealed a significantly greater total neuronal volume (Figure 2G, *p* = 0.0286) in the treated cultures than in the control cultures.

To further study the influence of EVs on neurons under normal conditions, their effects on spontaneous neuronal activity were evaluated. To achieve this goal, the neurons were cultured on MEA plates for 4 weeks after predifferentiation to achieve the mature functional stage as previously described (Figure 2A, (Hyvärinen et al. 2019)). In these cultures, at 72 h, EV‐treated neurons exhibited slightly but significantly higher LDH activity compared with control neurons (Figure 2D, *p* = 0.0057). However, compared with the effect of ionomycin, the positive control for cytotoxicity, which resulted in a 4‐fold increase in LHD activity (data not shown), the increase caused by EVs was considered minor. The baseline activity in both groups prior to EV exposure and 24 h after EV treatment is shown in Figure 2H as representative raster blots. Comparisons were performed against the baseline measurements in both groups. The mean firing rate did not differ after 24 h between the control and EV‐treated groups (Figure 2I), and similar results were observed throughout the 96‐h follow‐up period (Figure 2J). The median bursting rate was 5.8 bursts/min in the control group and 8.8 bursts/min in the EV‐treated group at the 24‐h timepoint, but the difference was not significant (Figure 2K, *p* = 0.5128). Compared with the baseline values, the control cultures showed a decrease in the bursting rate at 24 h (58% of the baseline), while the bursting rate remained at the baseline level in the EV‐treated group (Figure 2L). At the 48‐ and 96‐h timepoints, however, the bursting behaviour was similar in both groups with respect to the baseline (Figure 2L). Thus, at the functional level, EVs did not affect the neuronal firing rate, but there was a trend towards increased neuronal bursting 24 h after EV treatment. Overall, EVs had a minor effect on neuronal morphology and functionality under normal conditions.

### 3.3 Hypoxia Does Not Increase the Neuronal Internalization of EVs but Affects Their Colocalization With Cellular Organelles

**FIGURE 3:**
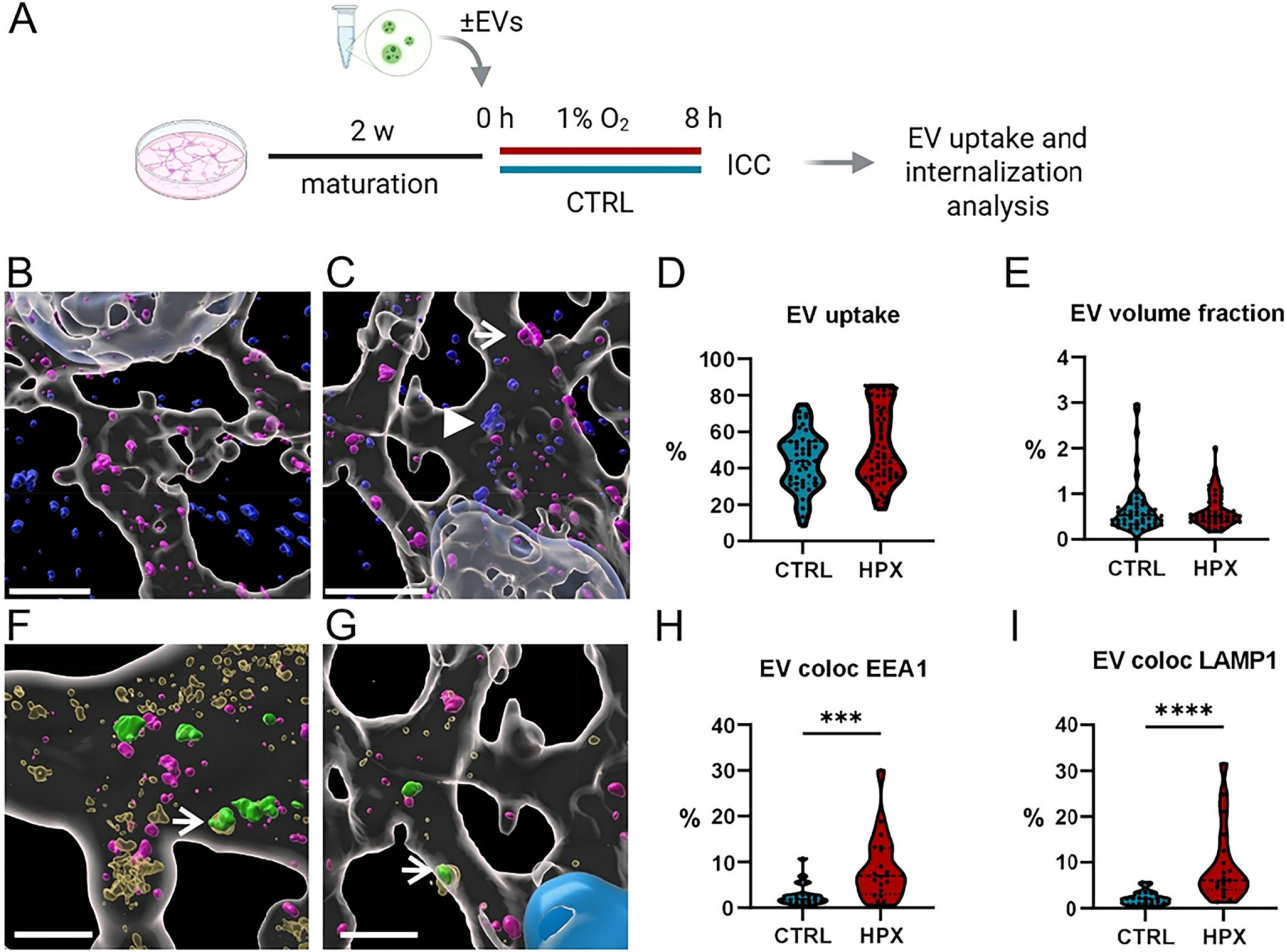
Internalization of EVs under hypoxic conditions. A) Timeline and experimental design. Created in BioRender. Harju, V. (2025) https://BioRender.com/m90izie. Reconstructions of neurons with 8 h of EV treatment under B) control or C) hypoxic conditions. The scale bars are 5 µm. In B and C, the EVs are blue when outside neurons (shown as a white surface) and violet when inside neurons. Notice in C the arrow indicating EV on the border of the neuron, calculated as internalized inside, and the arrowhead indicating EV outside the neuron, here beneath the transparent neuronal surface reconstruction. D) Quantification of the EV uptake under control and hypoxic conditions. E) Quantification of the total EV volume fraction inside neuronal surfaces under control and hypoxic conditions. The EV uptake results are from two experiments; each point represents one image (*n* = 52) taken from fifteen control and fourteen hypoxic cell cultures. The Mann‒Whitney U test revealed no significant differences in uptake. F) Reconstruction of EV colocalization with EEA1. Neuronal surface is shown in white, EEA1 in yellow; EVs inside neurons not colocalized with EEA1 are shown in violet, and EVs colocalized with EEA1 are shown in green, as indicated by the arrow. The scale bar is 3 µm. G) Reconstruction of EV colocalization with LAMP1. Neuronal surface is shown in white, LAMP1 in yellow; EVs inside neuron not colocalized with LAMP1 are shown in violet, and EVs colocalized with LAMP1 are shown in green, as indicated by the arrow. The scale bar is 3 µm. H) Quantification of EVs colocalized with EEA1 under control and hypoxic conditions. I) Quantification of EVs colocalized with LAMP1 under control and hypoxic conditions. The colocalization results are from two experiments, each point represents one image (*n* = 19–21) taken from nine individual control or eight individual hypoxic cell cultures. Statistical significances were calculated with the Mann‒Whitney U test, ***p < 0.0005, ****p < 0.0001. The results in D, E, H and I are presented as truncated violin plots, extending from minimum to maximum values, with dashed lines showing each quartile and dots showing individual data points. Exact *p* values are shown in Table S1. The numbers of samples and the numbers of EV surfaces included in the colocalization results are shown in Table S2. EV = extracellular vesicle, HPX = hypoxia, DIV = days in vitro, ICC = immunocytochemical staining.

The effects of hypoxia on EV internalization into neurons and colocalization with cellular organelles were evaluated at the 8‐h time point (Figure 3A). According to the ICC‐stained and reconstructed images, the degree of internalization was similar in both the control and hypoxic cultures (Figure 3B,C). The quantification of EV uptake revealed that on average, 44% of the EVs were internalized under the control condition and 46% were internalized under the hypoxic conditions, and the difference was not significant (Figure 3D, *p* = 0.0642). Additionally, the volume fraction of EVs of the total neuronal volume did not differ between conditions, being 0.53% under the control condition and 0.51% under the hypoxic condition (Figure 3E, *p* = 0.6911). Close‐up reconstructions of ICC staining with early endosomes (Figure 3F) and lysosomes (Figure 3G) under the hypoxic condition clearly revealed the colocalization of EVs with both organelles. Under the hypoxic condition, EVs showed significantly greater colocalization with EEA1 than under the control condition, with a colocalization rate of 1.8% in the control and 7.0% in the hypoxia group (Figure 3H, *p* = 0.0006). Colocalization with LAMP1 was also significantly greater under hypoxia than the control condition (6.1% vs. 2.1%, Figure 3I, *p* < 0.0001).

### 3.4 Neuronal Spiking and Bursting Are Affected by Hypoxia

**FIGURE 4:**
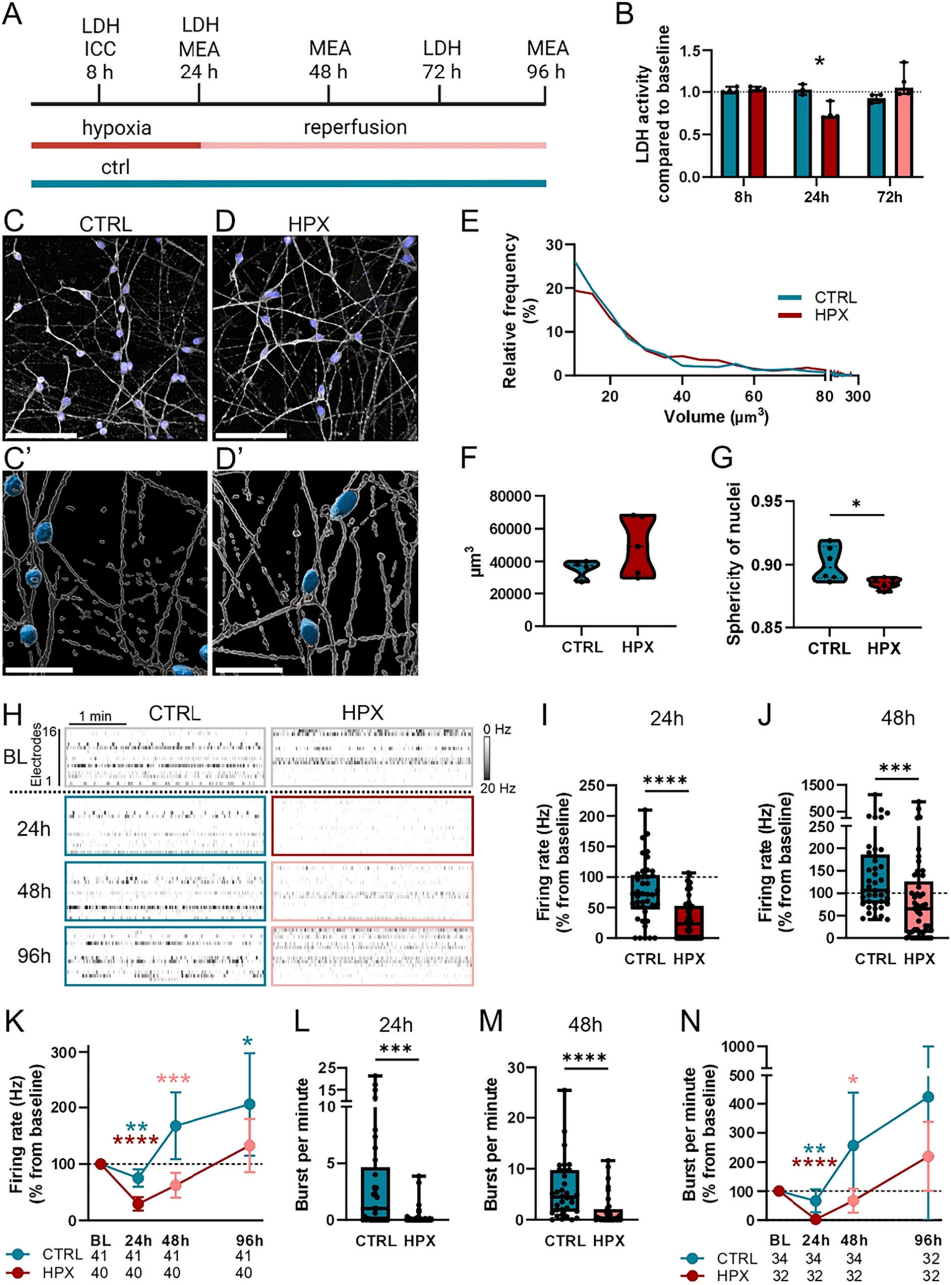
Neuronal morphology, viability and functionality under hypoxic conditions. A) Timeline and experimental design. Created in BioRender. Harju, V. (2025) https://BioRender.com/2yed8ce. B) LDH activity under control and after 8–24 h under hypoxic conditions and after 72 h (24 h hypoxia + 48 h reperfusion) compared with the baseline. Results are from one experiment, each sample (*n* = 4) pooled from two wells, *p < 0.05. The results are shown as median ± 95% CI. Statistical significances were calculated with the Kruskal‒Wallis test with Dunn's multiple comparisons. C) Staining and C’) reconstruction of neuronal surfaces under control condition. D) Staining and D’) reconstruction of neuronal networks after 8 h under hypoxia. In C, C’, D and D’, nuclei were stained with DAPI (blue), and neurons were co‐labelled with MAP‐2 + βtub_III_ (white/grey). The scale bars in C&D are 100 µm and those in C’&D’ are 50 µm. E) Frequency of neuronal surface volumes under control condition and after 8 h of hypoxia. F) The volumes of neuronal surfaces under control condition and after 8 h in hypoxia. G) The sphericity of neuronal nuclei under control condition and after 8 h in hypoxia. The volume and sphericity results are from one experiment; each point represents one image (*n* = 4–5) taken from two individual control and three individual hypoxic cell cultures. The results in F and G are presented as truncated violin plots, extending from minimum to maximum values, with dashed lines showing each quartile and dots showing individual data points. The Mann‒Whitney U test revealed no significant differences. H) Representative raster plots of neuronal activity under control condition and in hypoxia‐treated well after 24 h of hypoxia, 24 h of reperfusion and 72 h of reperfusion. I) Mean firing rate after 24 h under control and hypoxic conditions, compared with baseline, ****p < 0.0001. J) Mean firing rate after 48 h under control condition and after hypoxic (24 h hypoxia + 24 h reperfusion) treatment, compared with baseline, ***p < 0.0005. K) Firing rate compared with baseline for four days following the hypoxic treatment, shown as the median ± 95% CI. Significant differences at each timepoint between each condition and its own baseline were calculated with the Wilcoxon signed‐rank test. L) Burst per minute under control condition and after 24 h of hypoxia. M) Burst per minute after 48 h under control condition and after hypoxic (24 h hypoxia + 24 h reperfusion) treatment. Statistical significances in I, J, L, M were calculated with the Mann‒Whitney U test. N) Burst per minute compared with baseline for four days following the hypoxic treatment, shown as the median ± 95% CI. Significant differences between each condition and its own baseline were calculated with the Wilcoxon signed‐rank test. The MEA results are from three individual experiments: *n* = 42 for control and *n* = 40 for hypoxia group at baseline. *p < 0.05, **p < 0.005, ***p < 0.0005, ****p < 0.0001. Exact *p* values are shown in Table S1. The numbers of samples for each comparison and timepoint are shown in Table S2. LDH = lactate dehydrogenase, ICC = immunocytochemical staining, MEA = microelectrode array, HPX = hypoxia, BL = baseline.

To investigate the effects of hypoxia on neurons, their morphology and functionality were analyzed at multiple time points (Figure 4A). Percentage of Cl‐Casp3‐positive cells was higher after 8 h in hypoxia compared to control condition (Figure S3). However, LDH activity remained unchanged after 8 h of hypoxia, but it decreased after 24 h compared with to control (*p* = 0.0495) and recovered to control level after 48 h of reperfusion (Figure 4B). For neuronal morphology analysis, the reconstructions (Figure 4C’,D’) of ICC‐stained (Figure 4C,D) neuronal networks after 8 h of hypoxia were compared to those of controls. The morphology of the networks was not seemingly altered, but in terms of the frequency of neuronal volumes, more small neuronal fragments were detected under the control condition than under hypoxia (Figure 4E). However, the total neuronal volume remained similar; the median volume was 37541 µm^3^ for the control group and 49 253 µm^3^ for the hypoxia group (Figure 4F, *p* = 0.4127). There was a small change in the sphericity of neuronal nuclei between conditions, with hypoxia group having decreased sphericity (Figure 4G, *p* = 0.01737).

To investigate the detailed effects of hypoxia on neuronal functionality, neuronal activity was measured after 24 h of hypoxia, followed by after 24 h and 72 h of reperfusion (Figure 4A). Neuronal spiking and bursting were significantly reduced after 24 h of hypoxia compared with the control. Spiking was reduced in hypoxic wells to 21.5%, whereas in control wells, it was reduced to only 71.5% of baseline (Figure 4I, *p* < 0.0001). The decreased activity in the control wells was due to the long duration (48 h) since the previous media change. The bursting rate decreased to 1.0 bursts/min in the control, whereas in the hypoxic wells, it significantly decreased to zero (Figure 4L, *p* = 0.0002). After 24 h of reperfusion, both spiking (65% vs. 106%, Figure 4J, *p* = 0.0002) and bursting (0.2 vs. 4.5 bursts/min, Figure 4M, *p* < 0.0001) remained significantly lower in the hypoxia‐treated wells than in the control wells. However, the functional activities of the neuronal networks were restored to baseline levels or even higher after 72 h of reperfusion, as the spiking rate of hypoxia‐treated wells was 105% of its baseline, and the bursting rate was 86% of its baseline at the 96‐h timepoint, with no significant difference from the baseline value for either parameter (Figure 4K,N). This pattern was also evident in the raster plots of neuronal activity measured at baseline and after hypoxia at 24, 48 (24 h reperfusion), and 96 h (72 h reperfusion) (Figure 4H). Thus, 24 h of hypoxia decreased neuronal functionality only acutely, after which activity was restored after 72 h of reperfusion.

### 3.5 EVs Have no Significant Acute Effect on Cytotoxicity, Neuronal Morphology or Functionality Under Hypoxic Conditions

**FIGURE 5:**
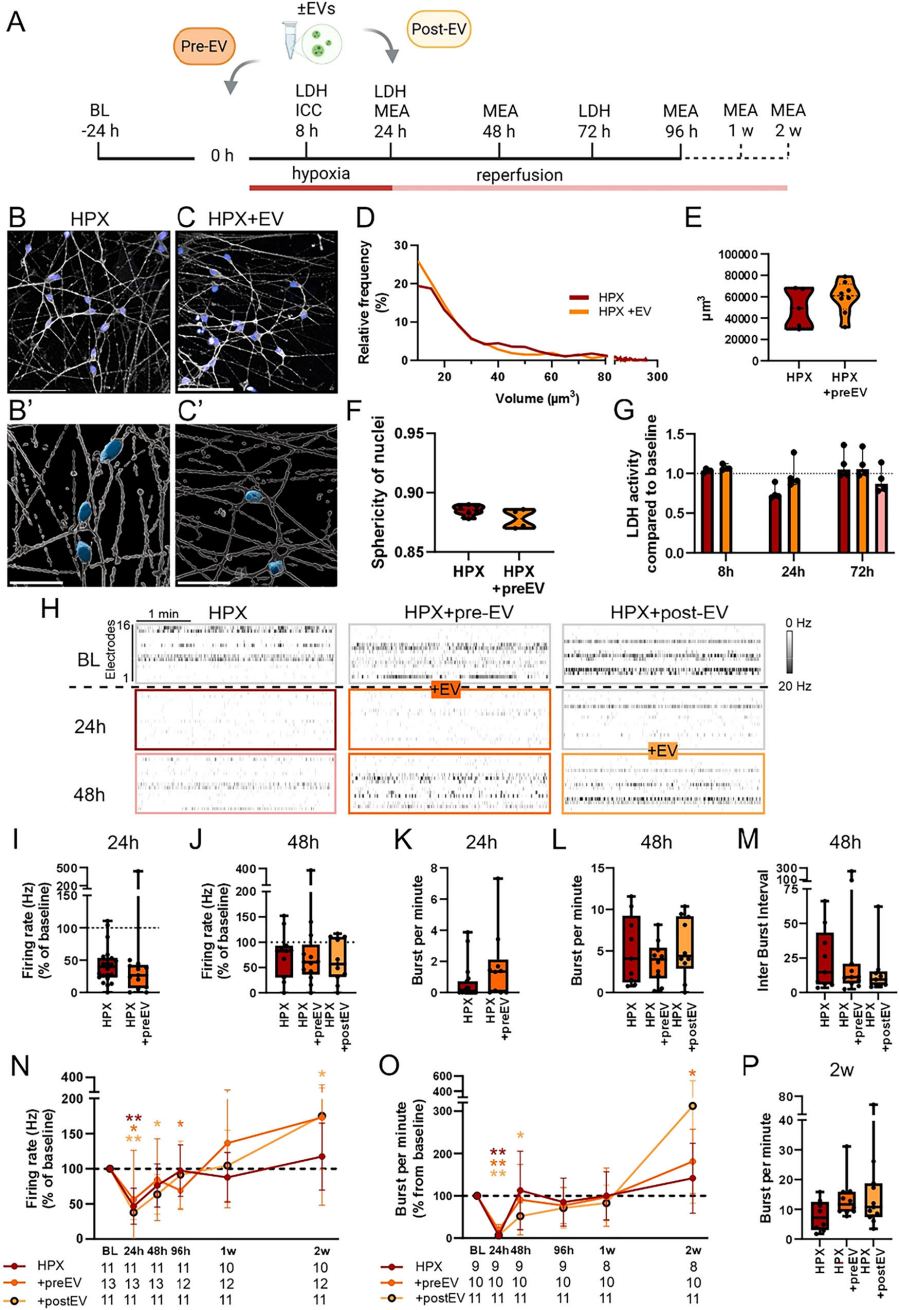
Acute and long‐term effects of EVs under hypoxic conditions. A) Timeline and experimental design. Created in BioRender. Harju, V. (2025) https://BioRender.com/ge3tu8f. B) Stained and B’) reconstructed images of neuronal surfaces under hypoxia. C) Stained and C’) reconstructed images of neuronal networks treated with EVs under hypoxia for 8 h. In B, B’, C and C’, nuclei were stained with DAPI (blue), and neurons were co‐labelled with MAP‐2 + βtub_III_ (white/grey). The scale bars in B&C are 100 µm, and those in B’&C’ are 30 µm. D) Frequency of neuronal surface volumes after 8 h in hypoxic conditions with or without EVs. E) Quantification of neuronal volumes and F) sphericity of neuronal nuclei after 8 h under hypoxic conditions with or without EVs. The volume and sphericity results are from one experiment; each point represents one image (*n* = 5–8) taken from three individual hypoxic cultures and four individual hypoxic cultures treated with EVs. The Mann‒Whitney U test revealed no significant differences. The results in E and F are presented as truncated violin plots, extending from minimum to maximum values, with dashed lines showing each quartile and dots showing individual data points. G) LDH activity under hypoxic conditions versus EV treatments. The results are from one experiment, and each sample (*n* = 4) pooled from two wells. The results are shown as the median ± 95% CI. The results of the Kruskal‒Wallis test with Dunn's multiple comparisons showed no significant differences. H) Representative raster plots of neuronal spiking activity before and after hypoxia without and with EV treatments. I) Acute mean firing rate after 24 h of hypoxia compared with baseline without and with pre‐EV treatment. J) Comparison of the mean firing rate after 48 h (24 h hypoxia + 24 h reperfusion) with baseline. K) Bursts per minute after 24 h of hypoxia without and with pre‐EV treatment. L) Bursts per minute after 48 h (24 h hypoxia + 24 h reperfusion) without and with pre‐/post‐EV treatment. M) Interburst interval after 48 h (24 h hypoxia + 24 h reperfusion) without and with pre‐/post‐EV treatment. N) Development of the mean firing rate during 2‐week period after hypoxia and hypoxia with pre‐ or post‐EV treatments. O) Development of the burst rate during 2‐week period hypoxia and hypoxia with pre‐ or post‐EV treatments. P) Burst rate 2 weeks after hypoxia and hypoxia with pre‐ or post‐EV treatments. Data in I‐L and P are presented as box plots with box borders for the 25th and 75th percentiles, whiskers for the minimum and maximum values, the middle line for the median and dots for the individual values. The Mann‒Whitney U test for comparisons between two groups and the Kruskal‒Wallis test for comparisons among three groups revealed no significant differences. Data in N and O are shown as the mean ± 95% CI. Significant differences between each condition and its own baseline were calculated with the Wilcoxon signed‐rank test, *p < 0.05, **p < 0.005. The MEA results are from one experiment: *n* = 9–11 wells. Exact *p* values are shown in Table S1. Number of samples in each comparison and timepoint are shown in Table S2. EV = extracellular vesicle, LDH = lactate dehydrogenase, ICC = immunocytochemical staining, MEA = microelectrode array, HPX = hypoxia, BL = baseline.

To investigate the effects of EV treatment on neuronal networks under hypoxia, EVs were administered either before (pre‐EV) or after (post‐EV) 24 h of hypoxia, and the effects were monitored both short‐term and long‐term (Figure 5A). After 8 h of hypoxia with or without EVs, the morphology of the neuronal networks appeared similar (Figure 5B,B’,C,C’). There was only a minor difference in the relative frequency of neuronal networks, as compared with hypoxia‐treated samples, EV‐treated samples had a slightly larger portion of smaller neuronal fragments (Figure 5D). Additionally, the volume of the neuronal surfaces or sphericity of nuclei did not significantly differ after 8 h of hypoxia with or without EVs (Figure 5E,F). However, the volume of the EV‐treated samples tended to increase, with a volume of 60 941 µm^3^, while the average volume of the hypoxia‐treated only samples was 49 253 µm^3^ (Figure 5E, *p* = 0.5237). LDH activity did not significantly differ between the EV‐treated group and the control group after 8 or 24 h of hypoxia or after 24 h of hypoxia followed by 48 h of reperfusion (Figure 5G).

The functional activity of the neurons was investigated before and after EV treatment. Representative raster blots (Figure 5H) revealed that both neuronal spiking and bursting decreased under hypoxia with or without EV treatment but recovered at least partly during reperfusion. After 24 h of hypoxia, the mean firing rate compared with baseline was 26% for pre‐EV‐treated samples and 40% for nontreated samples (Figure 5I, *p* = 0.1689). After 24 h of reperfusion, the firing rate was 83% of the baseline in the nontreated group, whereas it was 61% and 57% in the pre‐ and post‐EV‐treated groups, respectively (Figure 5J, *p* = 0.7125). The burst rates did not significantly change after 24 h of hypoxia or 24 h of reperfusion (Figure 5K,L, *p* = 0.0893 and *p* = 0.6717, respectively), even though the number of bursts was 1.4 bursts/min for pre‐EV‐treated wells compared with 0 for nontreated wells after 24 h of hypoxia (Figure 5K). The burst rate increased to 4.0–4.4 bursts/min in all groups after 24 h of reperfusion (Figure 5L). However, the interburst intervals tended to be shorter in the EV‐treated wells (9 sec in the post‐EV group and 11 sec in the pre‐EV group compared with 15 sec in the nontreated group) after 24 h of reperfusion (Figure 5M, *p* = 0.7141). Overall, there were no significant effects of EVs on neuronal cytotoxicity, morphology or functionality under hypoxic conditions after short‐term follow‐up.

### 3.6 Long‐term Effects of EV Treatment Tend to Increase Activity After 2 Weeks of Reperfusion

To elucidate the long‐term effects of EVs, the activity of neuronal networks was monitored for two weeks after hypoxia and EV treatment. After 24 h of hypoxia, all the groups exhibited significantly less activity, in terms of both spiking and bursting (Figure 5N, O, *p* values shown in Table S1). In the post‐EV treatment group, neuronal spiking and bursting were significantly lower after 24 h of reperfusion with EVs than at baseline (Figure 5N,O, *p* = 0.042 and *p* = 0.0098, respectively). In the pre‐EV treatment group, neuronal spiking decreased at 96 h (Figure 5N, *p* = 0.0425), but bursting remained at the baseline level (Figure 5O). Both neuronal spiking and bursting returned to baseline levels one week after the follow‐up (Figure 5N,O). However, at two weeks posttreatment, additional changes were observed. In networks subjected to hypoxic treatment alone, neuronal spiking and bursting remained close to baseline levels. In contrast, networks treated with hypoxia and EVs exhibited increased activity, with mean firing rates of 173% in the pre‐EV group and 175% in the post‐EV group compared with baseline, and the post‐EV group showing a significant difference from baseline (Figure 5N, *p* = 0.0186). The increase in the burst rates of neuronal networks in hypoxia‐treated wells was 141% of the baseline 2 weeks after treatment, whereas it was 181% in the pre‐EV‐treated group (*p* = 0.0488) and 312% in the post‐EV‐treated group (Figure 5O, *p* = 0.083). The number of bursts per min at two weeks post‐treatment was 7.2 in the hypoxia‐treated neuronal networks, whereas it was higher, although not significantly so, at 11.7 and 10.7 in the pre‐ and post‐EV‐treated groups, respectively (Figure 5P). Thus, the longer follow‐up time revealed possible long‐term effects of EVs on neuronal function, particularly the increase in neuronal activity 2 weeks after EV treatment.

## 4 Discussion

Here, the internalization of platelet‐derived EVs by hiPSC‐derived neurons is shown for the first time. Internalization and colocalization with cell organelles were evaluated in detail under both control and hypoxic conditions. The EVs were not cytotoxic, and their effects on the morphology and functionality of neurons were studied, revealing only a minor increase in neuronal volume. In addition, a hypoxic model of ischaemic stroke was established using functional neuronal cultures. The effects of hypoxic injury on neuronal networks were evaluated in detail at the morphological and functional levels, and both acute decreases in neuronal activity and recovery of activity during the reperfusion period were reported. Finally, potential treatments involving platelet‐derived EVs and their effects on neuronal networks under hypoxia were studied, both in short‐term and long‐term. We showed unique long‐term effects of the addition of EVs to the hypoxic neuronal network.

To elucidate the possible therapeutic effects of EVs, their contact with and internalization by neurons need to be studied in more detail. There are, however, challenges such as EV labelling, imaging at high enough resolution and 3D analysis (Verweij et al. 2021; Welsh et al. 2024). In our study, we utilized 3D imaging and detailed particle‐level image analysis with Imaris software, as also performed previously with mouse‐derived EVs and cells (Pantazopoulou et al. 2023). We used a fluorescent CFSE label incorporated inside the EVs and a staining protocol in which Triton X was replaced with saponin to ensure reliable imaging of EV internalization. The internalization of human platelet‐derived EVs into human neurons has not been previously reported; however, their internalization into naïve human aortic endothelial cells and HUVECs has been reported (Happonen et al. 2016). Our results revealed decreasing EV uptake of 64%–47% between 1 and 8 h by neurons under naïve conditions. Previous studies have shown saturation of the uptake of red blood cell‐derived EVs by HeLa cells before 1 h (Suutari et al. 2016) and the uptake of rat MSC‐derived EVs by neural stem cells (NSCs) after 2 h (Otero‐Ortega et al. 2020). We quantified the 0.3%–4% EV volume ratio to the neuronal volume, being slightly less than in a previous study reporting a volume ratio of ∼2%–15% of mouse brain‐derived EVs in mouse primary microglia and astrocytes (Pantazopoulou et al. 2023). Next, we studied the internalization of EVs under hypoxia. After 8 h of hypoxia, EV uptake into neurons was not altered significantly compared with that in control cultures. EV uptake studies in vitro under hypoxia are rare; however, increased uptake of glioblastoma‐derived EVs was reported in human glioblastoma cells after 6 h (Cerezo‐Magaña et al. 2021). Taken together, these results show constant uptake of human platelet‐derived EVs by human neurons under naïve and hypoxic conditions. Additionally, the uptake may be further enhanced by the modification of EV properties (Liu et al. 2023) or by targeted neuronal activation (Wang et al. 2025) for therapeutic purposes.

EVs can be internalized by multiple uptake mechanisms, but mainly endocytosis (Mulcahy et al. 2014), in which they are processed by a sequence of cellular organelles. Here, the colocalization of EVs with cellular organelle markers, for example, EEA1, an early endosome marker, and LAMP1, a lysosome marker, decreased between 1 and 8 h under naïve conditions, from 12% to 3% and from 11% to 2%, respectively. Compared to control condition, eight hours of hypoxia increased significantly both the colocalization with EEA1 from 1.8% to 7.0%, and with LAMP1 from 2.1% to 6.1%. Previously, the percentage of mouse brain‐derived EVs colocalized with LAMP1 was reported to be less than 1% in naïve primary microglia and astrocytes (Pantazopoulou et al. 2023); however, the analysis methods might not be directly comparable. Thus, human platelet‐derived EVs are internalized by human neurons and their internalization via endolysosomal pathway increases under hypoxic conditions.

The neuronal network volume increased 8 h after addition of EVs, in line with previous studies showing EVs from various sources to increase axonal length (Liu et al. 2020; Marzano et al. 2019). LDH activity slightly increased at 72 h after EV treatment, indicating cellular stress and/or increased metabolic activity rather than direct cytotoxicity as Cl‐Casp3 and LIVE/DEAD staining's did not show any changes. To study neuron‐specific behaviour and responses to different exposures, MEAs are excellent tools because they can be used noninvasively for the detection of neuronal network functionality in vitro (Pelkonen et al. 2021). Here, EV treatment did not significantly alter neuronal activity at the acute 96‐h follow‐up compared with the control cultures and their respective baseline recordings. Nevertheless, at the first time point (24 h), there was a slight decrease in neuronal activity under control conditions, which was not observed in EV‐treated cultures. The effects of EVs on neuronal functionality have rarely been studied; however, one study using human astrocyte‐derived EVs reported increased activity of hiPSC‐derived cortical neurons when measured with a patch‐clamp at the single‐neuron level (Chun et al. 2021). In conclusion, platelet‐derived EVs can cause minor morphological and cellular stress‐related changes in naïve human neurons; however, they do not have any detrimental effect on neuronal viability or functionality.

To model ischaemic stroke in vitro, 30 min to 24 h of insult with 1%–2% oxygen has been utilized previously (Cerina et al. 2024; Liu et al. 2020; Otero‐Ortega et al. 2020; Voogd et al. 2023). Here, we report that 8 h of hypoxia with 1% oxygen is not sufficient to cause cytotoxic or morphological changes in hiPSC‐derived neuronal cultures; thus, we instead used an insult duration of 24 h in 1% oxygen, which we have shown in an OGD model to induce neuronal damage under both 2D and 3D culture conditions (Juntunen et al. 2020; Räsänen et al. 2022). While 8 h of hypoxia caused a minor increase in apoptotic cell proportion, the 24 h of hypoxia caused a slight but significant decrease in LDH activity, which returned to baseline levels after 24 h of reperfusion. This might indicate a minor switch in cell death from basal level of necrosis in cell culture to apoptotic death during hypoxia. A decrease in LDH activity can be caused also by the reduced metabolic activity of neurons under hypoxic conditions. Indeed, functionally the neurons reacted to the 24 h of hypoxia, as indicated by decreased neuronal firing rates that continued after 24 h of reperfusion but returned to baseline levels after 48 h of reperfusion. The bursting rate decreased to almost to zero after 24 h of hypoxia but returned to baseline levels after 48 h of reperfusion. Similar decreases in neuronal activity patterns have been shown 24 h after exposure to 2% oxygen in hiPSC‐derived neurons and after 24 h of reperfusion (Pires Monteiro et al. 2021); however, no longer follow‐up periods have been reported. Thus, 24 h of hypoxia with 1% oxygen caused a reduction in neuronal activity that was recoverable after 48 h of reperfusion. The results of the cytotoxicity, morphological and functional analyses indicate that hiPSC‐based neuronal cultures have high plasticity and that spontaneous recovery is possible after acute hypoxia, resembling the injury in penumbra area.

From clinically validated human blood samples, it is possible to obtain platelets that have been shown to have positive outcome in in vivo models of stroke, such as increase in angiogenesis and neuroregeneration (Burnouf and Walker 2022; Leiter and Walker 2019; Rivera et al. 2016), and to extract EVs for therapeutic purposes (Aatonen et al. 2014). Here, platelet‐derived EVs were given either pre‐ or post‐hypoxia. No differences in neuronal morphology or LDH levels were detected between hypoxic and EV‐pretreated cultures after 8 h. LDH values did not differ between hypoxic and pretreated cultures at 24 h or between any group, including post‐treated EV cultures, after 48 h of reperfusion. Previously, hNSC‐derived EVs were shown to increase axonal length after hypoxia/reperfusion injury (Liu et al. 2020), and mouse NSC‐EVs to decrease neuronal cell death after OGD, as assessed by LDH release (Sun et al. 2019). A previous study revealed acutely increased neuronal activity in nontreated mouse brain slices by platelets (Kopeikina et al. 2020). Additionally, EVs from multiple hESC‐derived cell sources preserved the activity of hESC‐derived neurons 48 h after 1 h of OGD treatment (Deng et al. 2018). In our experiments, neuronal functionality, for example, firing and bursting rates, decreased in both the hypoxic and pretreated EV groups after 24 h of hypoxia; however, the number of bursts was slightly greater in the pretreated EV group. After 48 h of reperfusion, there were no differences between the hypoxia, pre‐ and post‐treated groups. Most likely, the differences in EVs, cells and hypoxic conditions used in various studies create variable results, highlighting the need for more focused in vitro work in this field.

Moreover, as modelling treatment for stroke, the long‐term effects of EVs should also be studied in vitro. Our long‐term follow‐up revealed that after one week, the baseline level functional activities were restored in all the groups, and interestingly, at 2 weeks, the activity in the post‐treated EV group was significantly greater than that at baseline, and the pre‐treated group showed greater activity than hypoxia‐treated cultures. These results suggest that EV treatment may modulate the long‐term activity recovery in neurons. There have been no other studies reporting long‐term effects on functionality with any treatment after hypoxia in vitro, as they have all focused on early time points up to 48 h. In vivo, MSC‐derived EVs enhanced functional recovery 28 d after stroke in mice (Doeppner et al. 2015; Otero‐Ortega et al. 2020), platelet‐derived microparticles alleviated behavioural deficits 20 d after stroke in rats (Hayon et al. 2012), and the effect of mesenchymal stromal cell‐derived EVs on post‐ischemic improvements in rats seemed to be higher than that of platelet‐EVs (Abuzan et al. 2025). This recovery is proposed to be due mainly to increased cell proliferation, angiogenesis and neurogenesis via growth factors, such as vascular endothelial growth factor and platelet‐derived growth factor, during the recovery period rather than a direct protective effect against the primary insult (Doeppner et al. 2015; Hayon et al. 2012, 2013; Otero‐Ortega et al. 2020). In vitro, no studies have reported the long‐term effects of EVs on neurons. However, better long‐term recovery after stroke could be achieved with naïve or engineered EVs via angiogenesis and neurogenesis (Mirzaahmadi et al. 2025) paving the way for more advanced stroke modelling in vitro.

Different kinds of in vitro set‐ups have been used for modelling ischaemic stroke, including rodent organotypic brain slices, brain organoids and 2D cell culture models, which still mostly rely on the use of rodent cells (Holloway and Gavins 2016). Here, we used a 2D cortical human neuronal model that contains both excitatory and inhibitory neurons and ∼10% astrocytes (Hyvärinen et al. 2019). To achieve more brain mimicry, the incorporation of microglia together with astrocytes would be advantageous because these cells are responsible for many neuroinflammation‐related events that occur after stroke (George and Steinberg 2015). To date, few multiculture models with human pluripotent stem cell‐derived neurons, astrocytes and microglia have been established, and improvements are still required, for example, medium optimization to support the microglia during long‐term culture (Guttikonda et al. 2021). To better model the therapeutic route, the integration of a BBB or neurovascular unit within models is suggested (Pang et al. 2024; Van Breedam and Ponsaerts 2022). However, simpler 2D neuronal models provide a more controlled environment and higher throughput, in which human‐derived cells are preferred (Holloway and Gavins 2016). As hiPSC‐derived neurons present quite developmentally young neurons (Mertens et al. 2018), they may have greater synaptic plasticity than mature neurons in the adult human brain, leading to faster and greater recovery than in vivo. Overall, a human cell‐based in vitro model should be established on the basis of the purpose of the study.

We showed the acute effect of hypoxia on hiPSC‐derived neurons along with long‐term spontaneous recovery. To model stroke treatment, we showed that EVs can be internalized by neurons and that they colocalize with early endosomes and lysosomes. EV treatment acutely affected the morphology and LDH activity of neurons but was associated with a better recovery of activity after hypoxia in the long term, with both pre‐ and post‐EV treatment. Finally, there is clinical potential for platelet‐derived EVs as neurological treatments, but more human cell‐based studies are needed to confirm their mode of action.

## Ethics Statement

The studied human pluripotent stem cell line has a supportive statement from the Ethics Committee of the Expert Responsibility area of Tampere University Hospital to be used in neuronal research (R20159). All donated blood products used for this research were treated anonymously, and donors had provided informed consent. The research was in accordance with the rules of the Finnish Supervisory Authority for Welfare and Health (Valvira, Helsinki, Finland). Research permission was also obtained from the local Blood Service Board (Finnish Red Cross Blood Service, Finland).

## Conflicts of Interest

The authors declare no conflict of interest.

## Supporting information




**Supplementary Figure 1**: Characterization of EVs. A) EV particle size distribution in volume and B) in concentration relative measured with NTA from two EV batches used in the study. C) Autofluorescence of EV‐free CFSE label control. D) Immunostained images of neuronal cell cultures with different concentrations of EVs: 10E+9, 10E+10 and 10E+11. DAPI stained the nuclei of the cells (blue), while neurons are co‐labelled with MAP‐2 + βtub_III_ (red) and EVs with green fluorescent protein (CFSE). Scale bar in C) and D) is 10 µm.
**Supplementary Figure 2**: Workflow of image analysis in Imaris software. A) EV uptake, original image from Imaris. EVs shown in green, nucleus in blue and neurons in red. Scale bar 5 µm. A’) Reconstruction of EVs as green surfaces and neurons as grey surfaces. A″) Showing filtering of EVs internalized inside neurons in pink and outside of neurons in blue. B) EV (green) colocalization with EEA1 (orange) after 8 h incubation with neurons (red) in control conditions, original image from Imaris. Scale bar in B)—B″’″) is 4 µm. B’) Reconstruction of neuronal surface with grey. B″) Reconstruction of EEA1 surfaces with yellow. B″’) Reconstruction of all EVs with green surfaces. B″″) Filtering non‐internalized EVs out. B″″’) Filtering EVs colocalized with EEA1 as red. C) EV (green) colocalization with LAMP1 (orange) after 8 h incubation with neurons (red) in control conditions, original image from Imaris. C’) Reconstruction of EVs as surfaces. Green surfaces are EVs colocalized with LAMP1 (yellow surfaces) and grey surfaces are not colocalized. C″) Reconstruction of EVs as spots. Green spots are EVs colocalized with LAMP1 (yellow surfaces) and grey spots are not colocalized. Scale bar in C), C’) and C″) is 3 µm.
**Supplementary Figure 3**: Viability of the cells. A) Immunostained and reconstructed image of neurons cultured 24 h in control condition. Cl‐Casp3‐positive nucleus shown in red, DAPI in blue and neurons in grey. B) Immunostained and reconstructed image of neurons incubated 24 h with EVs. Cl‐Casp3‐positive nucleus shown in red, EVs in green, DAPI in blue and neurons in grey. C) Percentage of cells with DAPI‐stained nucleus colocalizing with Cl‐Casp3. D) Number of live cells analyzed from LIVE/DEAD staining. E) Number of dead cells analysed from LIVE/DEAD staining. F) Immunostained and reconstructed image of neurons cultured 8 h in control condition. Cl‐Casp3‐positive nucleus shown in red, DAPI in blue and neurons in grey. G) Immunostained and reconstructed image of neurons cultured 8 h in hypoxia. Cl‐Casp3‐positive nucleus shown in red, DAPI in blue and neurons in grey. H) Percentage of cells with DAPI‐stained nucleus colocalizing with Cl‐Casp3. Scale bar in A), B), F), G) is 100 µm. The results in C, D, E and H are presented as truncated violin plots, extending from minimum to maximum values, with dashed lines showing each quartile and dots showing individual data points. Significances are calculated with the Mann‐Whitney test, *p < 0.05.
**Supplementary Table 1**: Statistical tests used, *p*‐value and medians or mean of each figure panel result. F = Figure number, P = Panel letter, KW = Kruskal‒Wallis, MW = Mann‒Whitney U, CTRL = control condition, EV = extracellular vesicle, BL = baseline, HPX = hypoxic condition.
**Supplementary Table 2**: Number of samples in each figure panel result and number of analyzed EV‐surfaces included in colocalization analysis. CTRL = control condition, EV = extracellular vesicle, BL = baseline, HPX = hypoxic condition.

## Data Availability

The data that support the findings of this study are available from the corresponding author upon reasonable request.
