# Supplementary material for: Human Platelet‐Derived Extracellular Vesicles Are Internalized by Human Induced Pluripotent Stem Cell‐Derived Neurons Under Control and Hypoxic Conditions
Source: J Extracell Biol. 2026 Jul 13;5(7):e70168. doi: 10.1002/jex2.70168 (PMC13364541; doi:10.1002/jex2.70168)
Supplement: Supplementary file 1 — Supplementary Figure 1: Characterization of EVs. A) EV particle size distribution in volume and B) in concentration relative measured with NTA from two EV batches used in the study. C) Autofluorescence of EV‐free CFSE label control. D) Immunostained images of neuronal cell cultures with different concentrations of EVs: 10E+9, 10E+10 and 10E+11. DAPI stained the nuclei of the cells (blue), while neurons are co‐labelled with MAP‐2 + βtubIII (red) and EVs with green fluorescent protein (CFSE). Scale bar in C) and D) is 10 µm. Supplementary Figure 2: Workflow of image analysis in Imaris software. A) EV uptake, original image from Imaris. EVs shown in green, nucleus in blue and neurons in red. Scale bar 5 µm. A’) Reconstruction of EVs as green surfaces and neurons as grey surfaces. A″) Showing filtering of EVs internalized inside neurons in pink and outside of neurons in blue. B) EV (green) colocalization with EEA1 (orange) after 8 h incubation with neurons (red) in control conditions, original image from Imaris. Scale bar in B)—B″’″) is 4 µm. B’) Reconstruction of neuronal surface with grey. B″) Reconstruction of EEA1 surfaces with yellow. B″’) Reconstruction of all EVs with green surfaces. B″″) Filtering non‐internalized EVs out. B″″’) Filtering EVs colocalized with EEA1 as red. C) EV (green) colocalization with LAMP1 (orange) after 8 h incubation with neurons (red) in control conditions, original image from Imaris. C’) Reconstruction of EVs as surfaces. Green surfaces are EVs colocalized with LAMP1 (yellow surfaces) and grey surfaces are not colocalized. C″) Reconstruction of EVs as spots. Green spots are EVs colocalized with LAMP1 (yellow surfaces) and grey spots are not colocalized. Scale bar in C), C’) and C″) is 3 µm. Supplementary Figure 3: Viability of the cells. A) Immunostained and reconstructed image of neurons cultured 24 h in control condition. Cl‐Casp3‐positive nucleus shown in red, DAPI in blue and neurons in grey. B) Immunostained and reconstructed image [file JEX2-5-e70168-s001.docx]

**Supplementary Material for Article**

**Human Platelet-Derived Extracellular Vesicles Are Internalized by Human Induced Pluripotent Stem Cell-Derived Neurons Under Control and Hypoxic Conditions**

Venla Harju^1^, Kai Härkönen^2^, Ulla Impola^2^, Saara Laitinen^2^, Susanna Narkilahti^1^

^1^ NeuroGroup, Faculty of Medicine and Health Technology, Tampere University, Tampere, Finland

^2^ Finnish Red Cross, Blood Service, Vantaa, Finland


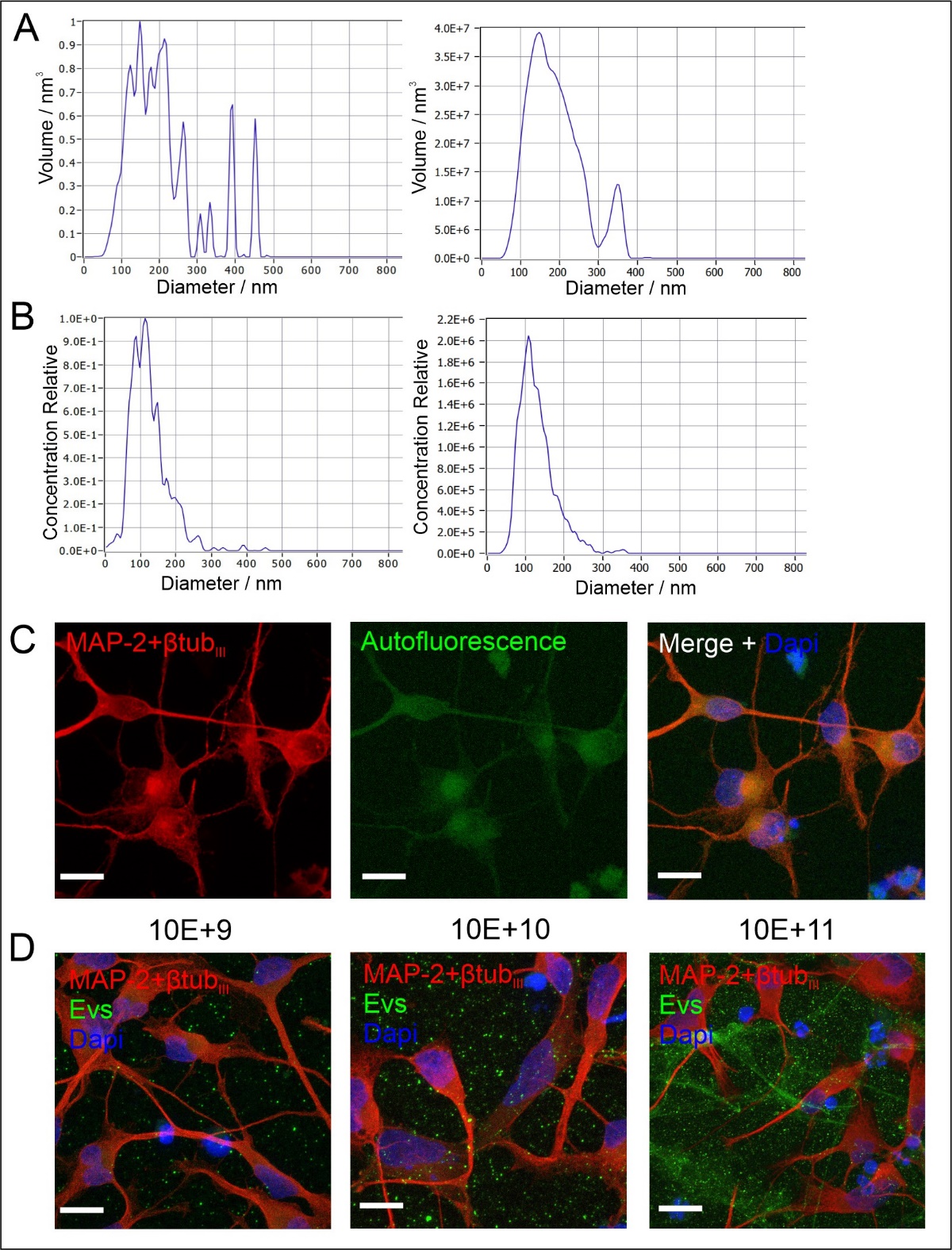


Supplementary Figure 1. Characterization of EVs. A) EV particle size distribution in volume and B) in concentration relative measured with NTA from two EV batches used in the study. C) Autofluorescence of EV-free CFSE label control. D) Immunostained images of neuronal cell cultures with different concentrations of EVs: 10E+9, 10E+10 and 10E+11. DAPI stained the nuclei of the cells (blue), while neurons are co-labelled with MAP-2 + βtub_III_ (red) and EVs with green fluorescent protein (CFSE). Scale bar in C) and D) is 10 µm.


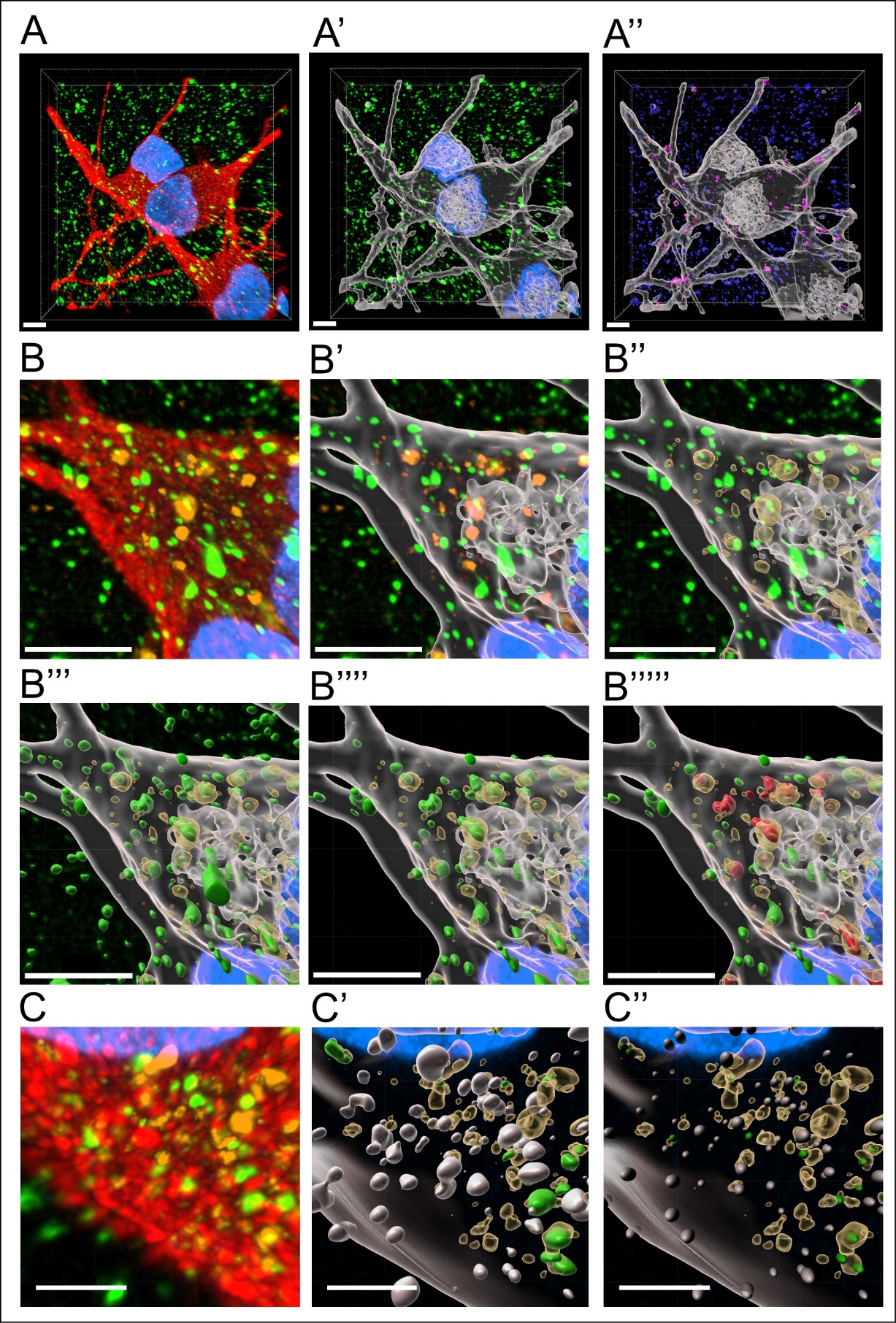


Supplementary Figure 2. Workflow of image analysis in Imaris software. A) EV uptake, original image from Imaris. EVs shown in green, nucleus in blue and neurons in red. Scale bar 5 µm. A’) Reconstruction of EVs as green surfaces and neurons as grey surfaces. A’’) Showing filtering of EVs internalized inside neurons in pink and outside of neurons in blue. B) EV (green) colocalization with EEA1 (orange) after 8 h incubation with neurons (red) in control conditions, original image from Imaris. Scale bar in B) - B’’’’’) is 4 µm. B’) Reconstruction of neuronal surface with grey. B’’) Reconstruction of EEA1 surfaces with yellow. B’’’) Reconstruction of all EVs with green surfaces. B’’’’) Filtering non-internalized EVs out. B’’’’’) Filtering EVs colocalized with EEA1 as red. C) EV (green) colocalization with LAMP1 (orange) after 8 h incubation with neurons (red) in control conditions, original image from Imaris. C’) Reconstruction of EVs as surfaces. Green surfaces are EVs colocalized with LAMP1 (yellow surfaces) and grey surfaces are not colocalized. C’’) Reconstruction of EVs as spots. Green spots are EVs colocalized with LAMP1 (yellow surfaces) and grey spots are not colocalized. Scale bar in C), C’) and C’’) is 3 µm.


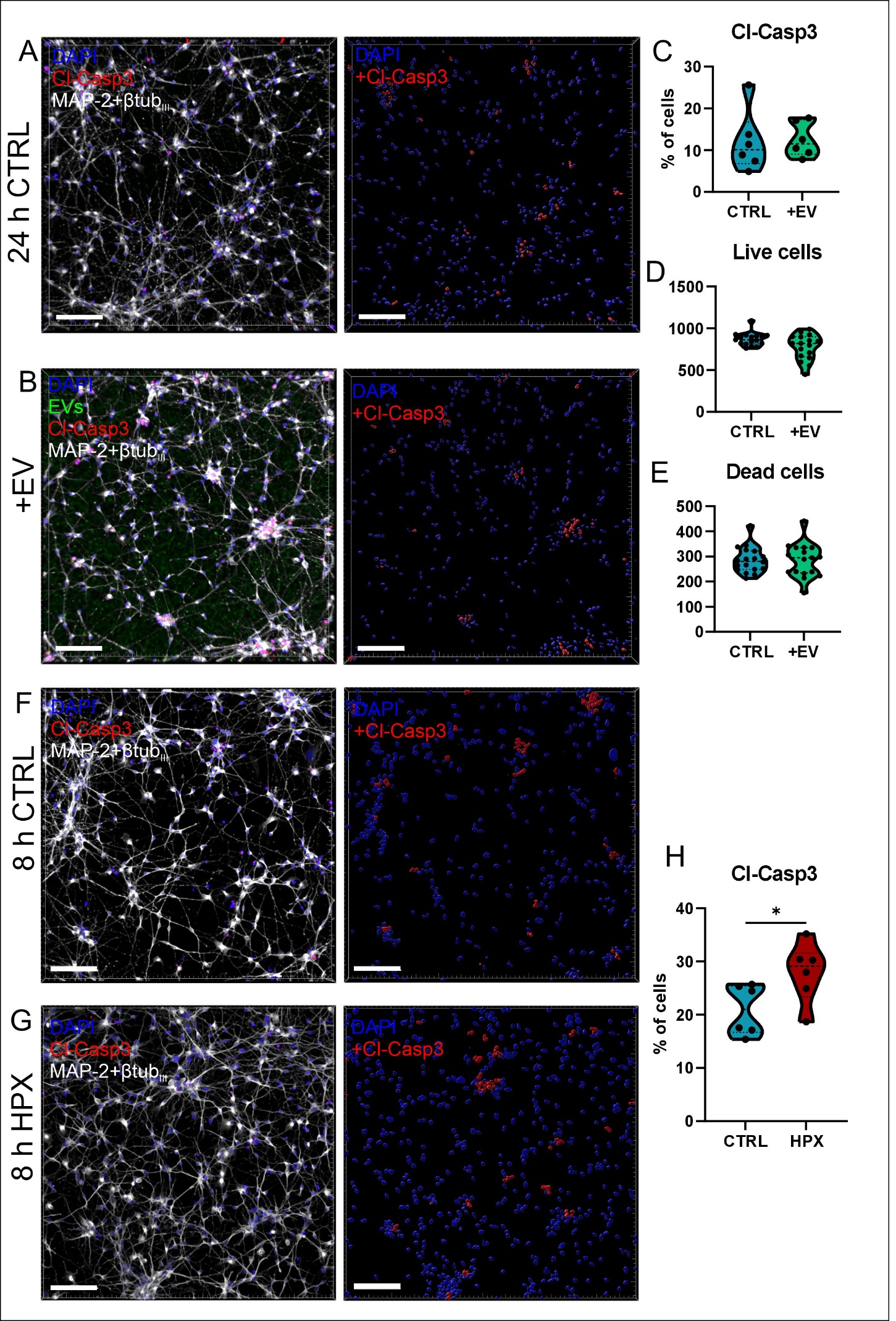


Supplementary Figure 3. Viability of the cells. A) Immunostained and reconstructed image of neurons cultured 24 h in control condition. Cl-Casp3-positive nucleus shown in red, DAPI in blue and neurons in grey. B) Immunostained and reconstructed image of neurons incubated 24 h with EVs. Cl-Casp3-positive nucleus shown in red, EVs in green, DAPI in blue and neurons in grey. C) Percentage of cells with DAPI-stained nucleus colocalizing with Cl-Casp3. D) Number of live cells analyzed from LIVE/DEAD staining. E) Number of dead cells analysed from LIVE/DEAD staining. F) Immunostained and reconstructed image of neurons cultured 8 h in control condition. Cl-Casp3-positive nucleus shown in red, DAPI in blue and neurons in grey. G) Immunostained and reconstructed image of neurons cultured 8 h in hypoxia. Cl-Casp3-positive nucleus shown in red, DAPI in blue and neurons in grey. H) Percentage of cells with DAPI-stained nucleus colocalizing with Cl-Casp3. Scale bar in A), B), F), G) is 100 µm. The results in C, D, E and H are presented as truncated violin plots, extending from minimum to maximum values, with dashed lines showing each quartile and dots showing individual data points. Significances are calculated with the Mann-Whitney test, **p* < 0.05.

Supplementary Table 1. Statistical tests used, *p*-value and medians or mean of each figure panel result. F = Figure number, P = Panel letter, KW = Kruskal‒Wallis, MW = Mann‒Whitney U, CTRL = control condition, EV = extracellular vesicle, BL = baseline, HPX = hypoxic condition

| **F** | **P** | **Comparison** | **Statistical test** | ***p*-value** | ***** | **Medians** | **Mean** |
| --- | --- | --- | --- | --- | --- | --- | --- |
| 1 | e | 1h vs 8h | MW | <0.0001 | **** | 63.81 vs 47.37 |  |
|  | f | 1h vs 8h | MW | 0.0097 | ** | 0.3192 vs 0.7666 |  |
|  | g | 1h vs 8h | MW | 0.0002 | *** | 12.08 vs 2.573 |  |
|  | h | 1h vs 8h | MW | 0.0027 | ** | 11.20 vs 2.222 |  |
| 2 | d | CTRL vs +EV at 8h | KW *p* = 0.0016  -> Dunn’s tests | 0.8202 |  | 1.01 vs 1.08 |  |
|  |  | CTRL vs +EV at 24h |  | 0.0668 |  | 1.03 vs 1.37 |  |
|  |  | CTRL vs +EV at 72h |  | 0.0057 | ** | 0.93 vs 1.19 |  |
|  | e | CTRL vs +EV | MW | >0.9999 |  | 0.8680 vs 0.8665 |  |
|  | g | CTRL vs +EV | MW | 0.0286 | * | 37541 vs 51140 |  |
|  | i | CTRL vs +EV | MW | 0.6673 |  | 74.51 vs 86.69 |  |
|  | j | BL vs CTRL at 24h | Wilcoxon | 0.1726 |  | 74.51% |  |
|  |  | BL vs CTRL at 48h |  | 0.7148 |  | 89.68% |  |
|  |  | BL vs CTRL at 96h |  | 0.9515 |  | 86.43% |  |
|  |  | BL vs +EV at 24h |  | 0.4263 |  | 86.69% |  |
|  |  | BL vs +EV at 48h |  | 0.5416 |  | 96.72% |  |
|  |  | BL vs +EV at 96h |  | 0.5016 |  | 90.37% |  |
|  | k | CTRL vs +EV | MW | 0.5128 |  | 5.83 vs 8.82 |  |
|  | l | BL vs CTRL at 24h | Wilcoxon | 0.3013 |  | 58.48% |  |
|  |  | BL vs CTRL at 48h |  | 0.8779 |  | 85.63% |  |
|  |  | BL vs CTRL at 96h |  | 0.6377 |  | 81.55% |  |
|  |  | BL vs +EV at 24h |  | 0.4238 |  | 100% |  |
|  |  | BL vs +EV at 48h |  | 0.9304 |  | 86.15% |  |
|  |  | BL vs +EV at 96h |  | 0.6069 |  | 63.42% |  |
| 3 | d | CTRL vs HPX | MW | 0.0642 |  | 43.93 vs 45.85 |  |
|  | e | CTRL vs HPX | MW | 0.6911 |  | 0.5266 vs 0.5112 |  |
|  | h | CTRL vs HPX | MW | 0.0006 | *** | 1.842 vs 6.962 |  |
|  | i | CTRL vs HPX | MW | <0.0001 | **** | 2.113 vs 6.087 |  |
| 4 | b | CTRL vs HPX at 8h | KW *p* = 0.0079  -> Dunn’s tests | > 0.9999 |  | 1.01 vs 1.04 |  |
|  |  | CTRL vs HPX at 24h |  | 0.0495 | * | 1.03 vs 0.72 |  |
|  |  | CTRL vs HPX at 72h |  | 0.1041 |  | 0.93 vs 1.05 |  |
|  | f | CTRL vs HPX | MW | 0.4127 |  | 37541 vs 49253 |  |
|  | g | CTRL vs HPX | MW | 0.0173 | * | 0.8980 vs 0.8855 |  |
|  | i | CTRL vs HPX | MW | < 0.0001 | **** | 71.48 vs 23.45 |  |
|  | j | CTRL vs HPX | MW | 0.0002 | *** | 106.4 vs 65.16 |  |
|  | k | BL vs CTRL at 24h | Wilcoxon | 0.0030 | ** | 71.48% |  |
|  |  | BL vs CTRL at 48h |  | 0.0840 |  | 106.4% |  |
|  |  | BL vs CTRL at 96h |  | 0.0404 | * | 107.1% |  |
|  |  | BL vs HPX at 24h |  | < 0.0001 | **** | 6.453% |  |
|  |  | BL vs HPX at 48h |  | 0.0003 | *** | 34.23% |  |
|  |  | BL vs HPX at 96h |  | 0.5272 |  | 105.4% |  |
|  | l | CTRL vs HPX 24h | MW | 0.0002 | *** | 1.02 vs 0 |  |
|  | m | CTRL vs HPX 48h | MW | < 0.0001 | **** | 4.46 vs 0.20 |  |
|  | n | BL vs CTRL at 24h | Wilcoxon | 0.0068 | * | 0% |  |
|  |  | BL vs CTRL at 48h |  | 0.0462 | * | 165.5% |  |
|  |  | BL vs CTRL at 96h |  | 0.3493 |  | 111.7% |  |
|  |  | BL vs HPX at 24h |  | < 0.0001 | **** | 0% |  |
|  |  | BL vs HPX at 48h |  | 0.0062 | ** | 0% |  |
|  |  | BL vs HPX at 96h |  | 0.4771 |  | 86.25% |  |
| 5 | e | HPX vs HPX+preEV | MW | 0.5237 |  | 49253 vs 60941 |  |
|  | f | HPX vs HPX+preEV | MW | 0.1810 |  | 0.8855 vs 0.8780 |  |
|  | g | HPX vs HPX+preEV at 8h | KW *p* = 0.0399  -> Dunn’s tests | > 0.9999 |  | 1.04 vs 1.06 |  |
|  |  | HPX vs HPX+preEV at 24h |  | 0.4461 |  | 0.72 vs 0.93 |  |
|  |  | HPX vs HPX+preEV at 72h |  | > 0.9999 |  | 1.05 vs 1.06 |  |
|  |  | HPX vs HPX+postEV at 72h |  | 0.6487 |  | 1.05 vs 0.87 |  |
|  | i | HPX vs HPX+preEV | MW | 0.1689 |  | 39.94 vs 26.47 |  |
|  | j | HPX | KW | 0.7125 |  | 82.65 |  |
|  |  | HPX+preEV |  |  |  | 60.78 |  |
|  |  | HPX+postEV |  |  |  | 56.94 |  |
|  | k | HPX vs HPX+preEV | MW | 0.0893 |  | 0.00 vs 1.35 |  |
|  | l | HPX | KW | 0.6717 |  | 4.08 |  |
|  |  | HPX+preEV |  |  |  | 4.00 |  |
|  |  | HPX+postEV |  |  |  | 4.38 |  |
|  | m | HPX | KW | 0.7141 |  | 14.66 |  |
|  |  | HPX+preEV |  |  |  | 11.07 |  |
|  |  | HPX+postEV |  |  |  | 9.33 |  |
|  | n | BL vs HPX at 24h | Wilcoxon | 0.0049 | ** | 36.57% | 46.72 |
|  |  | BL vs HPX at 48h |  | 0.1230 |  | 82.65% | 76.14 |
|  |  | BL vs HPX at 96h |  | 0.4648 |  | 88.74% | 97.33 |
|  |  | BL vs HPX at 1w |  | 0.4922 |  | 83.45% | 87.74 |
|  |  | BL vs HPX at 2w |  | 0.6250 |  | 106.8% | 117.4 |
|  |  | BL vs HPX+preEV at 24h |  | 0.0212 | * | 26.47% | 56.23 |
|  |  | BL vs HPX+preEV at 48h |  | 0.0803 |  | 60.78% | 84.36 |
|  |  | BL vs HPX+preEV at 96h |  | 0.0425 | * | 56.23% | 68.91 |
|  |  | BL vs HPX+preEV at 1w |  | 0.9097 |  | 94.30% | 136.5 |
|  |  | BL vs HPX+preEV at 2w |  | 0.0923 |  | 121.6% | 172.9 |
|  |  | BL vs HPX+postEV at 24h |  | 0.0010 | ** | 43.31% | 37.80 |
|  |  | BL vs HPX+postEV at 48h |  | 0.0420 | * | 56.94% | 63.45 |
|  |  | BL vs HPX+postEV at 96h |  | 0.4648 |  | 61.15% | 91.42 |
|  |  | BL vs HPX+postEV at 1w |  | 0.8984 |  | 70.72% | 104.5 |
|  |  | BL vs HPX+postEV at 2w |  | 0.0186 | * | 131.2% | 175.2 |
|  | o | BL vs HPX at 24h | Wilcoxon | 0.0039 | ** | 0% | 6.11 |
|  |  | BL vs HPX at 48h |  | 0.8203 |  | 52.03% | 112.7 |
|  |  | BL vs HPX at 96h |  | 0.3008 |  | 55.04% | 84.98 |
|  |  | BL vs HPX at 1w |  | 0.8438 |  | 129.9% | 99.79 |
|  |  | BL vs HPX at 2w |  | 0.2500 |  | 144.6% | 141.3 |
|  |  | BL vs HPX+preEV at 24h |  | 0.0020 | ** | 20.05% | 18.96 |
|  |  | BL vs HPX+preEV at 48h |  | 0.4316 |  | 44.29% | 90.64 |
|  |  | BL vs HPX+preEV at 96h |  | 0.1934 |  | 55.69% | 76.65 |
|  |  | BL vs HPX+preEV at 1w |  | 0.6953 |  | 53.38% | 96.66 |
|  |  | BL vs HPX+preEV at 2w |  | 0.0488 | * | 168.5% | 180.8 |
|  |  | BL vs HPX+postEV at 24h |  | 0.0010 | ** | 2.44% | 6.62 |
|  |  | BL vs HPX+postEV at 48h |  | 0.0098 | * | 38.44% | 51.83 |
|  |  | BL vs HPX+postEV at 96h |  | 0.2061 |  | 34.25% | 71.11 |
|  |  | BL vs HPX+postEV at 1w |  | 0.4648 |  | 97.55% | 82.67 |
|  |  | BL vs HPX+postEV at 2w |  | 0.0830 |  | 146.5% | 312.1 |
|  | p | HPX | KW | 0.2096 |  | 7.17 | 7.85 |
|  |  | HPX+preEV |  |  |  | 11.69 | 13.55 |
|  |  | HPX+postEV |  |  |  | 10.74 | 16.49 |

Supplementary Table 2. Number of samples in each figure panel result and number of analyzed EV-surfaces included in colocalization analysis. CTRL = control condition, EV = extracellular vesicle, BL = baseline, HPX = hypoxic condition

| **Figure** | **Panel** | **Treatment** | **Number**  **of samples** | **Number of analyzed EV-surfaces included in colocalization analysis** |
| --- | --- | --- | --- | --- |
| 1 | e | 1h | 45 |  |
|  |  | 8h | 43 |  |
|  | f | 1h | 47 |  |
|  |  | 8h | 44 |  |
|  | g | 1h | 22 | 3774 |
|  |  | 8h | 18 | 6080 |
|  | h | 1h | 19 | 7702 |
|  |  | 8h | 21 | 8569 |
| 2 | d | CTRL 8h | 4 |  |
|  |  | +EV 8h | 4 |  |
|  |  | CTRL 24h | 3 |  |
|  |  | +EV 24h | 4 |  |
|  |  | CTRL 72h | 4 |  |
|  |  | +EV 72h | 4 |  |
|  | e | CTRL | 6 |  |
|  |  | +EV | 6 |  |
|  | f, g | CTRL | 4 |  |
|  |  | +EV | 4 |  |
|  | i | CTRL | 14 |  |
|  |  | +EV | 14 |  |
|  | j | CTRL at 24h-96h | 14 |  |
|  |  | +EV at 24h-96h | 14 |  |
|  | k | CTRL | 12 |  |
|  |  | +EV | 13 |  |
|  | l | CTRL at BL | 12 |  |
|  |  | +EV at BL | 13 |  |
|  |  | CTRL at 24h | 12 |  |
|  |  | +EV at 24h | 13 |  |
|  |  | CTRL at 48h | 12 |  |
|  |  | +EV at 48h | 13 |  |
|  |  | CTRL at 96h | 11 |  |
|  |  | +EV at 96h | 12 |  |
| 3 | d, e | CTRL | 52 |  |
|  |  | HPX | 52 |  |
|  | h | CTRL | 21 | 14909 |
|  |  | HPX | 20 | 4916 |
|  | i | CTRL | 19 | 5523 |
|  |  | HPX | 19 | 5990 |
| 4 | b | CTRL | 4 |  |
|  |  | HPX | 4 |  |
|  | e, f | CTRL | 4 |  |
|  |  | HPX | 5 |  |
|  | g | CTRL | 6 |  |
|  |  | HPX | 6 |  |
|  | i | CTRL | 41 |  |
|  |  | HPX | 51 |  |
|  | j | CTRL | 41 |  |
|  |  | HPX | 45 |  |
|  | k | CTRL at BL | 35 |  |
|  |  | HPX at BL | 40 |  |
|  |  | CTRL at 24h | 35 |  |
|  |  | HPX at 24h | 40 |  |
|  |  | CTRL at 48h | 35 |  |
|  |  | HPX at 48h | 40 |  |
|  |  | CTRL at 96h | 35 |  |
|  |  | HPX at 96h | 40 |  |
|  | l | CTRL | 33 |  |
|  |  | HPX | 43 |  |
|  | m | CTRL | 34 |  |
|  |  | HPX | 32 |  |
|  | n | CTRL at BL | 22 |  |
|  |  | HPX at BL | 23 |  |
|  |  | CTRL at 24h | 22 |  |
|  |  | HPX at 24h | 23 |  |
|  |  | CTRL at 48h | 22 |  |
|  |  | HPX at 48h | 23 |  |
|  |  | CTRL at 96h | 22 |  |
|  |  | HPX at 96h | 23 |  |
| 5 | d, e | HPX | 5 |  |
|  |  | HPX+preEV | 8 |  |
|  | f | HPX | 6 |  |
|  |  | HPX+preEV | 4 |  |
|  | g | HPX | 4 |  |
|  |  | HPX+preEV | 4 |  |
|  |  | HPX+postEV | 4 |  |
|  | i | HPX | 22 |  |
|  |  | HPX+preEV | 13 |  |
|  | j | HPX | 11 |  |
|  |  | HPX+preEV | 13 |  |
|  |  | HPX+postEV | 11 |  |
|  | k | HPX | 19 |  |
|  |  | HPX+preEV | 10 |  |
|  | l | HPX | 9 |  |
|  |  | HPX+preEV | 10 |  |
|  |  | HPX+postEV | 10 |  |
|  | m | HPX | 9 |  |
|  |  | HPX+preEV | 11 |  |
|  |  | HPX+postEV | 9 |  |
|  | n | HPX at BL | 11 |  |
|  |  | HPX+preEV at BL | 13 |  |
|  |  | HPX+postEV at BL | 11 |  |
|  |  | HPX at 24h | 11 |  |
|  |  | HPX+preEV at 24h | 13 |  |
|  |  | HPX+postEV at 24h | 11 |  |
|  |  | HPX at 48h | 11 |  |
|  |  | HPX+preEV at 48h | 13 |  |
|  |  | HPX+postEV at 48h | 11 |  |
|  |  | HPX at 96h | 11 |  |
|  |  | HPX+preEV at 96h | 12 |  |
|  |  | HPX+postEV at 96h | 11 |  |
|  |  | HPX at 1w | 10 |  |
|  |  | HPX+preEV at 1w | 12 |  |
|  |  | HPX+postEV at 1w | 11 |  |
|  |  | HPX at 2w | 10 |  |
|  |  | HPX+preEV at 2w | 12 |  |
|  |  | HPX+postEV at 2w | 11 |  |
|  | o | HPX at BL | 9 |  |
|  |  | HPX+preEV at BL | 10 |  |
|  |  | HPX+postEV at BL | 11 |  |
|  |  | HPX at 24h | 9 |  |
|  |  | HPX+preEV at 24h | 10 |  |
|  |  | HPX+postEV at 24h | 11 |  |
|  |  | HPX at 48h | 9 |  |
|  |  | HPX+preEV at 48h | 10 |  |
|  |  | HPX+postEV at 48h | 11 |  |
|  |  | HPX at 96h | 9 |  |
|  |  | HPX+preEV at 96h | 10 |  |
|  |  | HPX+postEV at 96h | 11 |  |
|  |  | HPX at 1w | 8 |  |
|  |  | HPX+preEV at 1w | 10 |  |
|  |  | HPX+postEV at 1w | 11 |  |
|  |  | HPX at 2w | 8 |  |
|  |  | HPX+preEV at 2w | 10 |  |
|  |  | HPX+postEV at 2w | 11 |  |
|  | p | HPX | 8 |  |
|  |  | HPX+preEV | 10 |  |
|  |  | HPX+postEV | 11 |  |
